# Identification of TUBB2A by quantitative proteomic analysis as a novel biomarker for the prediction of distant metastatic breast cancer

**DOI:** 10.1186/s12014-020-09280-z

**Published:** 2020-05-24

**Authors:** Dongyoon Shin, Joonho Park, Dohyun Han, Ji Hye Moon, Han Suk Ryu, Youngsoo Kim

**Affiliations:** 1 Department of Biomedical Sciences, Seoul National University College of Medicine, 103 Daehakro, Seoul, 30380 Korea; 2 Interdisciplinary Program for Bioengineering, Seoul National University College of Engineering, Seoul, Korea; 3 Biomedical Research Institute, Seoul National University Hospital, 101 Daehakro, Seoul, Korea; 4 Department of Pathology, Seoul National University Hospital, 101 Daehakro, Seoul, 03080 Korea

**Keywords:** Distant metastatic breast cancer, Formalin-fixed paraffin-embedded (FFPE) tissue, Biomarkers, Tandem mass tag (TMT), Quantitative proteomics

## Abstract

**Background:**

Metastasis of breast cancer to distal organs is fatal. However, few studies have identified biomarkers that are associated with distant metastatic breast cancer. Furthermore, the inability of current biomarkers, such as HER2, ER, and PR, to differentiate between distant and nondistant metastatic breast cancers accurately has necessitated the development of novel biomarker candidates.

**Methods:**

An integrated proteomics approach that combined filter-aided sample preparation, tandem mass tag labeling (TMT), high pH fractionation, and high-resolution MS was applied to acquire in-depth proteomic data from FFPE distant metastatic breast cancer tissues. A bioinformatics analysis was performed with regard to gene ontology and signaling pathways using differentially expressed proteins (DEPs) to examine the molecular characteristics of distant metastatic breast cancer. In addition, real-time polymerase chain reaction (RT-PCR) and invasion/migration assays were performed to validate the differential regulation and function of our protein targets.

**Results:**

A total of 9441 and 8746 proteins were identified from the pooled and individual sample sets, respectively. Based on our criteria, TUBB2A was selected as a novel biomarker candidate. The metastatic activities of TUBB2A were subsequently validated. In our bioinformatics analysis using DEPs, we characterized the overall molecular features of distant metastasis and measured differences in the molecular functions of distant metastatic breast cancer between breast cancer subtypes.

**Conclusions:**

Our report is the first study to examine the distant metastatic breast cancer proteome using FFPE tissues. The depth of our dataset allowed us to discover a novel biomarker candidate and a proteomic characteristics of distant metastatic breast cancer. Distinct molecular features of various breast cancer subtypes were also established. Our proteomic data constitute a valuable resource for research on distant metastatic breast cancer.

## Background

Breast cancer is one of the most prevalent and lethal cancers in women worldwide [1]. In particular, its annual incidence—currently 17 million cases—is increasing at an alarming rate [2, 3]. There are approximately 232,000 new cases of invasive breast cancer each year in the US, and approximately 40,000 women die each year from the disease; furthermore, roughly 90% of these deaths are caused by the most malignant form of breast cancer: distant metastatic breast cancer [2, 4]. Distant metastatic breast cancer, which preferentially metastasizes to distal organs, such as the bone, liver, lung, and brain, has a poor prognosis [5, 6]. In addition, this type of breast cancer causes various complications at the affected sites, such as pericardial effusion, pleural effusion, bone fracture, hypercalcemia, and red blood cell anemia, which worsens survival outcomes [7–9].

Distant metastatic breast cancer is assessed, based on various factors, such as tumor size, lymphovascular invasion, histological grade, nodal involvement, and hormone receptor status—all of which are independent risk factors for distant metastatic breast cancer [10–13]. Among these factors, breast cancer molecular subtypes are associated with various patterns of distant metastatic spread and related to differences in survival outcomes [10, 14]. For instance, the most widely known molecular subtypes, such as the luminal A, luminal B, HER2, and basal-like (triple-negative) groups, have site-specific, cumulative metastatic incidence rates, demonstrating substantial differences in the distant metastatic behavior of and overall survival between breast cancer subtypes [10].

Although various risks and molecular characteristics of distant metastatic breast cancer have been established, the prediction and diagnosis of distant metastasis in breast cancer with molecular biomarkers remain largely unexamined [4–6, 10–13]. Thus, characterizing the molecular signatures that are associated with distant metastasis using omics-based approaches, such as genomics, transcriptomics, and proteomics, might identify previously overlooked biomarker candidates.

Many genomic or transcriptomic studies have examined the molecular characteristics of distant metastatic breast cancer—for instance, genes that are associated with lung, brain, and bone metastasis from breast tumor [15–18, 20, 21]. In addition, genetic signatures that predict distant metastasis in breast cancer have been established through genomic profiling [19]. However, given the relatively low correlation between gene expression and protein expression, it is difficult to assume that the tendencies in genomic data will translate fully to proteomic data without verification [22, 23]. Similarly, considering that transcriptomic and proteomic data have a moderate correlation, the molecular characteristics of the transcriptome could not perfectly represent those of the proteome [24–26]. In the case of breast cancer, recent large dataset-based proteomic approaches have reported an intermediate correlation between the breast tumor proteome and the corresponding transcript levels [27, 28]. Furthermore, a recent report has described a low correlation between proteomes and transcriptomes in human breast cancer tissues, suggesting that a proteomic approach to human BC tissues could complement a transcriptomic method [29].

Although proteomic studies have been performed for various diseases, including breast cancer, none has investigated the overall characteristics of distant metastatic breast cancer [29–37, 44]. Proteomic research is expected to provide greater insight into the pathogenesis of distant metastatic breast cancer, generating novel information about the molecular features of distant metastasis—for example, by discovering novel protein biomarkers for the prediction or diagnosis of distant metastatic breast cancer. Thus, an in-depth proteomic analysis is important for yielding valuable resources in distant metastatic breast cancer—data that have not been found in genomic and transcriptomic analyses.

Recent advances in mass spectrometry (MS)-based proteomics have accelerated the development of high-throughput techniques for proteomic quantification [38, 39]. In addition, a tandem mass tag (TMT)-based strategy has facilitated relative protein quantification by comparing the reporter ion intensities that are obtained by MS/MS. Because this approach can quantify thousands of proteins precisely with high sensitivity, TMT-based techniques have been used widely to generate substantial datasets [40–43]. With a 6-plex TMT quantification technique, in combination with high-resolution MS, we constructed an in-depth proteomic map of distant metastatic breast cancer.

In this study, we hypothesized that in-depth proteomic data would supply important proteins to profile the molecular signatures of distant metastatic breast cancer. Using our proteomic techniques, we identified by far the largest number of proteins from FFPE distant and nondistant metastatic breast cancer tissues. Furthermore, we determined important protein targets to validate distant metastatic potential of breast cancer. The function of these targets was determined using several approaches, including RT-PCR and invasion/migration assays.

Through our criteria to narrow down the important proteins, we discovered a novel protein biomarker candidate differentially expressed in distant metastatic breast cancer. Furthermore, we examined the distinct biological functions of distant metastatic breast cancer between molecular subtypes. In summary, we have proposed the first protein biomarker candidate that potentially be able to distinguish distant metastasis, derived from primary breast tumors using FFPE tissue samples. We performed the initial examination of its molecular features at the protein level, providing insights into the pathogenesis of distant metastatic breast cancer.

## Methods

### Materials and reagents

Sodium dodecyl sulfate (SDS) and Trizma base were purchased from USB (Cleveland, OH), and sequencing-grade modified trypsin was purchased from Promega Corporation (Madison, WI). Dithiothreitol (DTT) and urea were obtained from AMRESCO (Solon, OH). POROS20 R2 beads were purchased from Applied Biosystems (Foster City, CA). High-purity (> 97%) mass spectrometry (MS)-grade ovalbumin was obtained from Protea (Morgantown, WV), and HLB OASIS columns were purchased from Waters (Milford, MA). Tandem mass tag (TMT) 6-plex isobaric reagents; a bicinchoninic acid (BCA) assay kit; LC/MS-grade solvents, such as acetone, acetonitrile (ACN), and water; and reducing agents, such as tris (2-carboxyethyl) phosphine (TCEP), were purchased from Thermo Fisher Scientific (Waltham, MA). All other reagents, if not noted otherwise, were obtained from Sigma-Aldrich (St. Louis, MO).

### Sample selection

All clinical samples were collected from the Department of Pathology, Seoul National University Hospital (Seoul, South Korea). The distant metastasis group (dis-meta) was defined as patients who developed distant metastasis with or without lymph node metastasis. The nondistant metastasis group (nondis-meta) comprised patients who were not diagnosed as having distant metastasis with or without lymph node metastasis. All clinical specimens were collected from 18 patients with dis-meta and 18 patients with nondis-meta. The 18 patients in each group were divided into 3 breast cancer molecular subtypes (HER2, TNBC, and luminal). Tissue samples for distant and nondistant metastatic breast cancer were derived from the primary breast tumor. Clinical information on the patient samples is detailed in Additional file 1: Table S1. All patients consented to participate in the study per institutional review board guidelines (IRB No.1612-011-811).

### Sample preparation of FFPE tissues for proteomic analysis

FFPE sections (10 μm) were incubated twice in xylene (Sigma-Aldrich, St. Louis, MO)—once each for 5 and 2 min—and then twice in 100% (v/v) ethanol for 90 s. The sections were then hydrated in 75% (v/v) ethanol for 90 s and distilled water for 90 s [33, 44]. Next, the tissues were scraped off the glass slides into microfuge tubes, after which protein extraction buffer (4% SDS; 0.3 M Tris, pH 8.5; 2 mM TCEP) was added. Following sonication, the samples were incubated at 100 °C for 2.5 h. Protein concentrations were measured using a bicinchoninic acid (BCA) reducing agent-compatible kit (Thermo Fisher Scientific, Waltham, MA).

Protein digestion was performed using a combination of acetone precipitation and filter-aided sample preparation (FASP) [45, 46]. Before the digestion step, 250 μg of extracted protein was precipitated with cold acetone at a buffer: acetone ratio of 1:5 and incubated at – 20 °C for 18 h. Next, the pellet was washed with 500 μl cold acetone, centrifuged at 15,000 rpm for 15 min, and air-dried for 1.5 h. The proteins that had precipitated were dissolved in 35 μl denaturation buffer (4% SDS and 100 mM DTT in 0.3 M TEAB pH 8.5).

After being heated at 100 °C for 35 min, the denatured proteins were loaded onto 30 kDa spin filters (Merck Millipore, Darmstadt, Germany). The buffer was exchanged 3 times with UREA solution (8 M UREA in 0.1 M TEAB, pH 8.5). After SDS was removed, cysteine residues were treated with alkylation buffer (50 mM IAA, 8 M UREA in 0.1 M TEAB, pH 8.5) for 1 h at room temperature in the dark. UREA buffer was exchanged with TEAB buffer (40 mM TEAB, pH 8.5). The proteins were digested with trypsin (enzyme-to-substrate ratio [w/w] of 1:50) and 4% ACN at 37 °C for 18 h. The digested peptides were eluted by centrifugation, and their concentrations were measured, based on the fluorescence emission of tryptophan at 350 nm, using an excitation wavelength of 295 nm [47]. The external standard sample, ovalbumin, was digested in the same manner.

### 6-Plex tandem mass tag (TMT) labeling

Because the number of samples exceeded that of the TMT channels, 2 independent TMT 6-plex labeling experiments—using a pooled sample set and individual sample set—were performed. Each TMT experiment consisted of 18 samples that were divided into 2 groups (dis-meta and non dis-meta). For the pooled sample set, equal amounts of 3 samples with identical molecular subtypes in each group were pooled, generating 6 pooled samples. Next, they were labeled with TMT 6-plex: 126-non dis-meta (HER2), 127-non dis-meta (TNBC), 128-non dis-meta (Luminal), 129-dis-meta (HER2), 130-dis-meta (TNBC), and 131-dis-meta (Luminal). At this step, several technical replicates of the sample sets were prepared. For the individual sample set, 18 individual patients were positioned in 3 TMT 6-plex sets: 126-non dis-meta (HER2), 127-non dis-meta (TNBC), 128-non dis-meta (Luminal), 129-dis-meta (HER2), 130-dis-meta (TNBC), and 131-dis-meta (Luminal). The detailed experimental workflow is described in Additional file 2: Fig. S1.

Prior to the TMT labeling step, 45 μg of each peptide sample was mixed with an equivalent volume of ovalbumin. Then, 40 mM TEAB buffer was added to each sample to equalize the volume. Next, TMT reagents were reconstituted in 110 μl anhydrous ACN. Each sample was labeled using 25 μl of the reconstituted TMT reagent. Then, 45 μl ACN was added in varying volumes to a final concentration of 30% and incubated at room temperature (25 °C) for 1.25 h. Hydroxylamine was added in various volumes to a concentration of 0.3% (v/v) to quench the reaction. TMT-labeled samples for each set were pooled at a ratio of 1:1. The pooled sample was lyophilized and desalted.

### Desalting and high-pH reversed-phase (HPRP) peptide fractionation

The TMT-labeled samples were desalted on an HLB OASIS column per the manufacturer’s instructions. High-pH reversed-phase (HPRP) peptide fractionation was performed on an Agilent 1260 bioinert HPLC instrument (Agilent, Santa Clara, CA) with an Agilent 300 Extended-C18 column (4.6 mm I.D × 15 cm long, 5-μm C18 particle). TMT-labeled peptide samples were prefractionated at a flow rate of 1 mL/min for 60 min on a linear gradient, which ranged from 5% to 40% ACN with 15 mM ammonium hydroxide. The sample was separated into 96 fractions, which were then assembled into 12 fractions. The 12 fractions were lyophilized and stored at − 80 °C before MS analysis.

### Sample preparation of breast cancer cells for proteomic analysis

MDA-MB-231 breast cancer cells were cultured in DMEM, and T47D cells were cultured in RPMI, containing 10% FBS and 1% penicillin and streptomycin. The cells were seeded in 75-cm^2^ culture plates. After a 24-h incubation at 37 °C with 5% CO_2_, the cells were scraped using a cell scraper and washed 3 times with 1 × PBS. The scraped cell pellets were centrifuged and washed again 3 times with 1 x PBS. The pellets were then transferred to microfuge tubes and mixed with protein extraction buffer (4% SDS; 0.3 M Tris, pH 7.5; 2 mM TCEP). Following sonication, the samples were incubated at 100 °C for 30 min. After protein extraction, the subsequent experimental procedures, such as protein digestion, TMT labeling, desalting, and peptide fractionation, were performed in the same manner as the FFPE tissues.

### Reversed-phase (RP)-nano LC–ESI–MS/MS analysis

The prefractionated peptides were analyzed on an LC–MS system with an Easy-nLC 1000 (Thermo Fisher Scientific, Waltham, MA) that was equipped with a nanoelectrospray ion source (Thermo Fisher Scientific, Waltham, MA) and coupled to a Q-Exactive mass spectrometer (Thermo Fisher Scientific, Waltham, MA), as described in our previous studies [45, 46]. The peptide samples were separated on a 2-column system, comprising a trap column (Thermo Fisher Scientific, 75 μm I.D. x 2 cm long, 3-μm Acclaim PepMap100 C18 beads) and an analytical column (Thermo Fisher Scientific, 75 μm I.D. x 50 cm long, 3-μm ReproSil-Pur C18-AQ beads). Lyophilized peptide samples were dissolved in Solvent A (0.1% formic acid water and 2% ACN) prior to injection.

The peptides were separated on a 180-min linear gradient, ranging from 6 to 26% Solvent B (100% ACN and 0.1% formic acid) for all peptide samples. The spray voltage was set to 2.2 kV in positive ion mode, and the heated capillary temperature was set to 320 °C. Mass spectra were collected in data-dependent acquisition (DDA) mode by top 20 method. Xcaliber (version 2.5) was used to set the mass spectrometer parameters as follows: mass range to 350–1650 m/*z*, resolution of 70,000 at 200 m/z for detected precursor ions, automatic gain control (AGC) at 3 x 10^6^, isolation window for MS2 at 1.2 m/z, automatic gain control (AGC) for MS2 at 2 x 10^5^, higher-energy collisional dissociation (HCD) scans at a resolution of 35,000, and normalized collision energy (NCE) of 32. The maximum ion injection time (maximum IT) for the full-MS and MS2 scans was 30 ms and 120 ms, respectively. Dynamic exclusion with an exclusion time of 40 s was used.

### MS data search

Proteome Discoverer, version 2.2 (Thermo Fisher Scientific, Waltham, MA) was used to search the resulting RAW files. The full-MS and MS/MS spectra search was conducted using the SEQUEST HT algorithm against a modified version of the Uniprot human database (December 2014, 88,717 protein entries; http://www.uniprot.org), which included chicken ovalbumin. The database search was performed using the target-decoy strategy. The search parameters were as follows: a precursor ion mass tolerance value of 20 ppm (monoisotopic mass); a fragment ion mass tolerance value of 0.02 Da (monoisotopic mass); full enzyme digest with trypsin (after KR/−) and up to 2 missed cleavages; static modification values of 229.163 Da for lysine residues and peptide N-termini for TMT labeling and 57.02 Da for cysteine residues with carbamidomethylation; and dynamic modification values of 42.01 Da for protein N-terminal acetylation, 0.984 Da for asparagine deamidation, and 15.99 Da for methionine oxidation.

A false discovery rate (FDR) of less than 1% at the peptide and protein levels was used as the confidence criteria. Proteins were quantified by computing reporter ion relative intensities with the “Reporter Ions Quantifier” node in Proteome Discoverer. The co-isolation threshold value was 70%. The mass spectrometry-based proteome data lists of all identified proteins and peptides have been deposited into ProteomeXchange (http://proteomecentral.proteomexchange.org) through the PRIDE partner repository: dataset identifier PXD016061 [48, 69–71].

### Quantification of protein abundance and statistical analysis

Protein levels were normalized, based on the ovalbumin content in each TMT channel. Fold-change values were calculated by dividing the average value of the normalized protein abundance in the dis-meta group by that of the non dis-meta group. Statistical analysis for the proteomic data was performed for the normalized protein levels using Perseus (version 1.5.8.5). Student’s t-test was used to identify differentially expressed proteins (DEPs) for selecting biomarker candidates that differentiate distant metastasis from nondistant metastasis of breast cancer. The statistical cutoff for the student’s t-test was a p-value < 0.05. In addition, ANOVA was used to determine DEPs for analyzing the molecular characteristics of distant metastatic breast cancer between molecular subtypes using bioinformatic tools. Specifically, 9 samples in each group were classified as HER2, TNBC, and luminal, resulting in 6 subtype groups (HER2 nondis-meta, TNBC nondis-meta, luminal nondis-meta, HER2 dis-meta, TNBC dis-meta, and luminal dis-meta). Next, the quantified proteins in these groups were analyzed to detect statistically significant proteins. The statistical cutoff for the ANOVA was p-value < 0.05. Receiver operating characteristic (ROC) analyses of biomarker performance were performed using MedCalc (version 12.5.0) and Prism (version 6.0).

### Bioinformatics analysis

The Gene Ontology (GO) of the proteins was classified using the DAVID bioinformatics tool (version 6.8). GO classification was assessed by Fisher’s exact test to obtain a series of p-values that were filtered, based on a statistical significance of 0.05. Canonical pathways and downstream biological functions were enriched by Ingenuity Pathway Analysis (IPA, QIAGEN, Redwood City, CA). The analytical algorithms in IPA were used to predict the downstream effects on known biological pathways and functions, based on the inputted list of DEPs. IPA allocates activation scores on activated or inhibited status to biological functions and pathways that underlie the quantitative values of proteins. Fisher’s exact test was used to acquire p-values, whereas the degree of activation was measured using Z-scores. The p-value cutoff was set to 0.05, and the predictive activation Z-score cutoff was set to a magnitude of 1.

### RNA extraction and real-time polymerase chain reaction (RT-PCR)

Total RNA was isolated from the following breast cancer cell lines using TRIzol (Invitrogen, Carlsbad, CA, USA) per the manufacturer’s instructions: MCF10A, MCF7, T47D, BT474, skBR3, MDA-MB-453, BT-20, MDA-MB-468, HCC70, HCC38, MDA-MB-157, MDA-MB-436, MDA-MB-231, and HS578T. Two micrograms of total RNA from each cell line was used for the reverse-transcription reaction. First-strand cDNA was synthesized by standard random priming with RNA inhibitor (Promega, Madison, WI) and Moleney murine leukemia virus reverse transcripts (Promega, Madison, WI). Following cDNA synthesis, target genes were amplified using specific primers and HIPI plus Master mix (ElpisBio, Daejeon, Korea).

### Cell lines and culture conditions for invasion and migration assays

The MDA-MB-231 and Hs578T cell lines were obtained from American Type Culture Collection (ATCC; Manassas, VA, USA) and the Korean Cell Line Bank (KCLB, Seoul, Korea), respectively. The cells were cultured in DMEM (Gibco, CA, USA), containing 10% fetal bovine serum (FBS; Invitrogen, Carlsbad, CA, USA) and 1% penicillin/streptomycin (Gibco, CA, USA). The cells were maintained at 37 °C in a humidified atmosphere of 95% air and 5% CO_2_ and screened periodically for mycoplasma contamination. Both cell lines were confirmed by DNA profiling of short tandem repeats (STRs) by the KCLB (Seoul, Korea).

### Small interfering RNA (siRNA) transfection

siRNAs that targeted LTF and TUBB2A and AccuTarget Negative Control siRNA were purchased from Bioneer (Daejeon, Korea). The siRNA sequences for LTF and TUBB2A were as follows: siLTF-1, 5′-GAGAUCAGACACUACCUU-3′; siLTF-2, 5′-CACACUGUUGAUGUAAUGA-3′; siTUBB2A-1,′-CUCAAGCAUGGUCUUUCA-3′; siTUBB2A-2, 5′-CACACUGUUGAUGUAAUGA-3′. Cells were transfected using Lipofectamine RNAiMAX (Invitrogen, Carlsbad, CA, USA) per the manufacturer’s instructions. After a 48-h incubation, silencing of LTF and TUBB2A was confirmed by measuring their respective mRNA levels.

### Cell migration and invasion assays

Quantitative cell migration and invasion were assessed using 24-well inserts (Corning Incorporated, NY, USA) with 8-μm pores according to the manufacturer’s instructions. In brief, for the transwell migration assay, transfected cells (5 × 10^4^ cells) were seeded into the upper chamber, and medium that contained 10% FBS was added to the lower chamber. After a 24-h incubation, the cells on the top of the membrane were removed using a cotton swab. The remaining migrant cells were washed with PBS, fixed in 4% paraformaldehyde, stained with 1% crystal violet for 10 min, and imaged and counted in 3 randomly selected fields under a microscope (Nikon, Tokyo, Japan). These experiments were performed in triplicate.

For the in vitro invasion assay, the upper wells of Boyden chambers were coated with 2 mg/ml of Matrigel (Corning Incorporated, NY, USA) at 37 °C in a 5% CO_2_ incubator for 2 h. The cells (1 × 10^5^ cells) were seeded into the upper chamber, and medium that contained 10% FBS was added to the lower chamber. The rest of the assay was performed as described above.

## Results

### Construction of distant metastatic breast cancer proteomic datasets

**Fig. 1:**
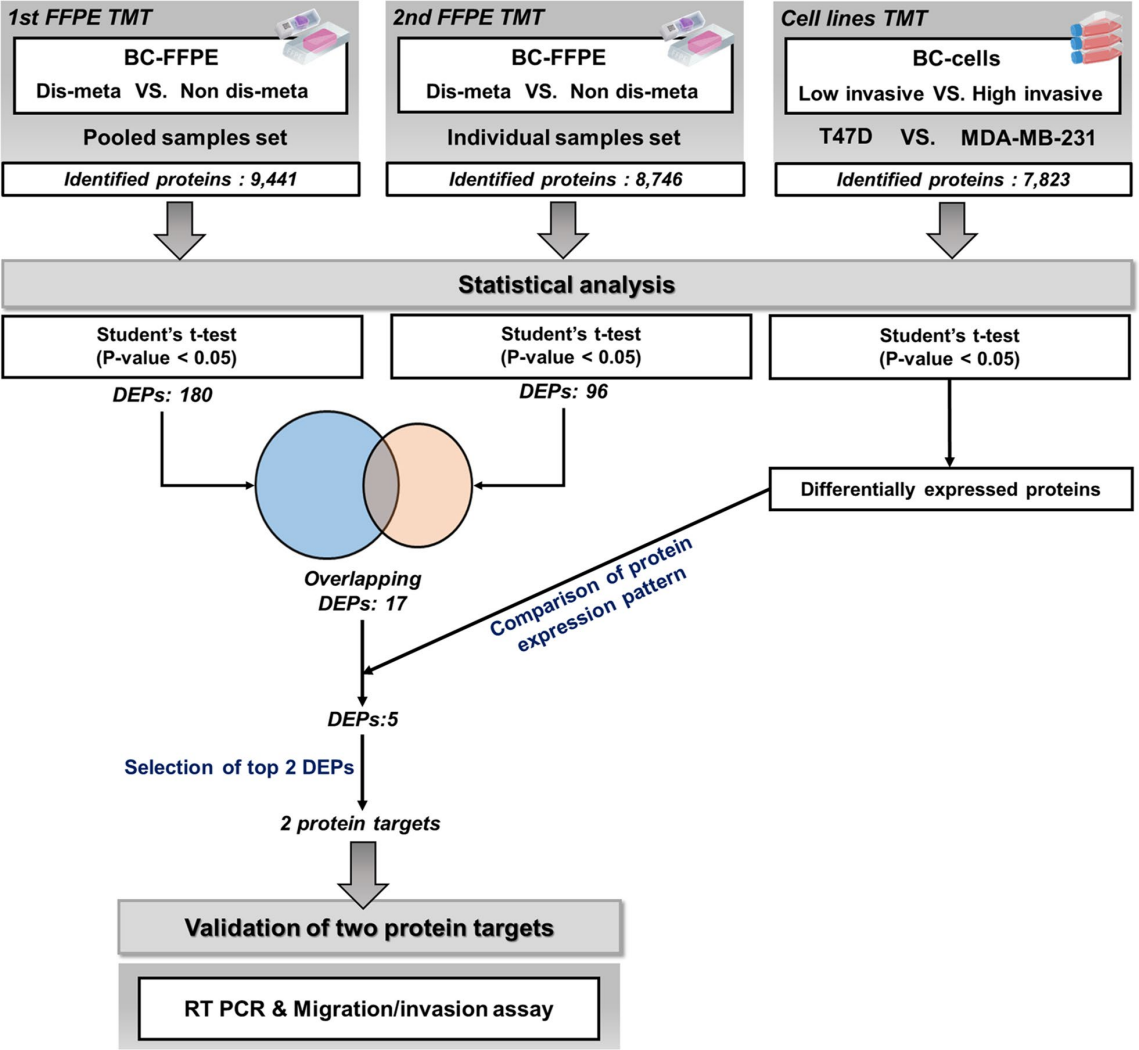
Schematic of overall proteomic results of the TMT-based proteomic analysis. Number of identified proteins; pooled sample set: 9441, individual sample set: 8746, and cell line set: 7823. Number of DEPs by statistical analysis and the steps for selection of protein targets. Validation phase of protein targets; real-time polymerase chain reaction (RT-PCR) and migration/invasion assay

In the pooled sample set, 9441 proteins were identified, and 7179 proteins were quantified across all samples. In the individual sample set, 8746 proteins were identified, and 6642 proteins were quantified in all samples (Fig. 1, Additional file 2: Fig. S2a). In addition, the number of identifications in each sample was calculated, resulting in a range from 7515 to 7798 identified proteins in the individual sample set and 8287 to 8309 proteins in the pooled sample set. Overall, the numbers of proteins in the samples of each sample set were similar (Additional file 2: Fig. S2b–c).

Our proteomic platform enabled us to perform an in-depth analysis of the distant metastatic breast cancer proteome, as evidenced by a dynamic range that spanned over 6 orders of magnitude (Additional file 2: Fig. S3). This comprehensive dataset included many established biomarkers for breast cancer, including the receptor tyrosine kinase erbB-2 (HER2), estrogen receptor (ESR1), progesterone receptor (PGR), and androgen receptor (AR). Notably, established protein biomarkers for metastatic breast cancer, such as EGFR, HSPD1, PRDX6, and TPM4, which are related to lymph node and regional metastasis, were also detected [50]. Moreover, this proteome encompassed most of the identified proteins in our previous study and included an additional 3757 and 3126 newly identified proteins in the pooled and individual sample sets, respectively (Additional file 2: Fig. S4) [44]. Consequently, our in-depth proteomic profiling generated a comprehensive dataset that is suitable for biomarker discovery and analysis with regard to determining the underlying mechanisms of distant metastasis in breast cancer. All identified proteins of each sample set are listed in Additional file 3: Table S2.

### Quality assessment of proteomic data

The multiplexing feature of the TMT-based strategy allowed us to examine the quantitative variation within and between our samples. Interbatch and intrabatch variation was assessed using an internal standard, ovalbumin. As a result, the interbatch and intrabatch normalization produced coefficients of variation of 4.17% and 6.7% in the pooled and individual sample sets, respectively (Additional file 2: Fig. S5a). Although the variation in non-normalized intensities reflected excellent reproducibility, a slight improvement in reproducibility was observed when the levels of proteins were normalized to ovalbumin (Additional file 2: Fig. S5b–c).

Next, correlation values were calculated to assess the variation between technical replicates in the pooled sample set. MS analysis of the pooled sample set showed excellent correlation, with Pearson’s correlation values ranging from 0.993 to 0.994 and averaging 0.993 (Additional file 2: Fig. S5d). In addition, the correlation between the quantitative levels of all samples was calculated to assess the variation across individual samples. MS analysis of the individual sample set revealed a wider range of correlation values than that of the pooled sample set, with Pearson’s correlation values ranging from 0.647 to 0.988 and averaging 0.927 (Additional file 2: Fig. S5e). One sample, a HER2 type in the non dis-meta group, had low correlation values when paired with other individual samples, resulting in a range of 0.647 to 0.778. Slight differences in protein abundance between individual samples were observed.

### Determination of protein targets to validate distant metastatic potential

To select important protein targets to verify distant metastatic potential of breast cancer, the quantified proteins in the BC FFPE tissues datasets (i.e., the pooled and individual sample sets) were examined separately by statistical analysis. For the proteomic datasets of BC FFPE tissues, student’s t-test was performed to determine differentially expressed proteins (DEPs) between the nondistant metastasis and distant metastasis groups. When a Benjamini–Hochberg false discovery rate (BH-FDR) cutoff of 0.05 was applied to the proteins in the pooled and individual sample sets respectively, however, none of the proteins in nondis-meta and dis-meta was significantly differentially expressed. Nonetheless, to determine protein targets for validation of distant metastatic breast cancer, alternative criteria were applied to the datasets.

The criteria were as follows: 1. The quantified proteins in our BC FFPE tissue proteomic datasets must pass a p-value (unadjusted for multiple comparison) cutoff of 0.05 by student’s t-test for determining DEPs in nondis-meta versus dis-meta. 2. Overlapping DEPs in both BC FFPE tissue datasets were selected. 3. Overlapping DEPs that were also identified in the BC cell line proteomic dataset and demonstrated a consistent expression pattern in all 3 datasets were selected. 4. Overlapping DEPs that passed a fold-change cutoff of 1.2 were selected. 5. The most highly up-regulated and down-regulated DEPs were selected. Therefore, DEPs that satisfied all of the requirements were selected as protein targets for validation of distant metastatic potential (Fig. 1).

Specifically, a total of 180 and 96 proteins were initially selected as DEPs by student’s t-test (p-value < 0.05) in the pooled and individual sample sets, respectively (Fig. 1, Additional file 4: Table S3). Next, overlapping proteins in DEPs of each sample set were selected.

As a result, 17 overlapping DEPs in both sets were selected. The results of the statistical analysis for these proteins are listed in Table 1. Of the 17 proteins, 5 (HSPA9, PSMB4, CTNNA1, XPO5, and PAFAH1B3) functioned in the growth, proliferation, metastasis, and recurrence of cancer [51–56]. Specifically, HSPA9 was associated with metastasis of hepatocellular carcinoma (HCC), and overexpression of HSPA9 increased the malignancy and aggressive behavior of HCC [51, 52]. Overexpression of PSMB4 increases cellular growth and the viability of breast cancer and ovarian cancer, leading to a poor prognosis [53, 54]. The deletion of CTNNA1 effects the loss of cell-to-cell adhesion, enhancing the growth and mobility of breast cancer cells [55]. XPO5 exports pre-miRNAs through the nuclear membrane to the cytoplasm and is thus important in breast cancer tumorigenesis [56]. PAFAH1B3 is a critical driver of the pathogenicity of breast cancer by inhibiting tumor-suppressing signaling lipids [72]. These 5 proteins were upregulated in our distant metastasis group, which we propose stimulate the distant metastatic potential of breast cancer.

**Table 1:** Detailed statistical analysis of 17 overlapping proteins

Protein name	Dis-meta vs non dis-meta in pooled sample set				Dis-meta vs non dis-meta in individual sample set				High invasive vs low invasive in cell lines set				Consistency of protein expression	Fold-change > 1.2
	*t* Test significance	*p* Value	Adjusted *p* value (BH FDR < 0.05)	Fold-change	*t* Test significance	*p* Value	Adjusted *p* value (BH FDR < 0.05)	Fold-change	*t* Test significance	*p* Value	Adjusted *p* value (BH FDR < 0.05)	Fold-change		
Glyceraldehyde-3-phosphate dehydrogenase	+	0.01308	1	1.211	+	0.03681	1	1.260	+	0.01580	0.02242	0.815	N	N
Tubulin beta-2A chain	+	0.01730	1	1.219	+	0.01980	1	1.298	+	0.00076	0.00173	2.329	Y	Y
Lactotransferrin	+	0.00000	9.269E − 09	0.581	+	0.02619	1	0.546	+	0.00529	0.00866	0.551	Y	Y
Stress-70 protein, mitochondrial	+	0.00251	0.85696	1.114	+	0.04264	1	1.160	+	0.00003	0.00019	0.742	N	N
Catenin alpha-1	+	0.04027	1	1.137	+	0.03017	1	1.189	+	0.00000	0.00003	0.473	N	N
Bifunctional purine biosynthesis protein PURH	+	0.03371	1	1.150	+	0.03078	1	1.180	+	0.00047	0.00119	0.710	N	N
Heterogeneous nuclear ribonucleoprotein H	+	0.04529	1	1.046	+	0.02779	1	1.111	N/D	N/D	N/D	N/D	N/D	N
Isoform 2 of Multifunctional protein ADE2	+	0.01657	1	1.149	+	0.02412	1	1.147	+	0.00184	0.00355	0.742	N	N
ADP/ATP translocase 3	+	0.01062	1	0.827	+	0.04718	1	0.833	+	0.00022	0.00068	1.416	N	N
Exportin-5	+	0.00720	1	1.177	+	0.03871	1	1.227	+	0.00359	0.00619	0.868	N	N
RNA-binding protein 39	+	0.04237	1	1.074	+	0.02435	1	1.119	+	0.00171	0.00334	0.916	N	N
Acyl-Coenzyme A dehydrogenase, C-4 to C-12 straight chain, isoform CRA_a	+	0.04319	1	0.922	+	0.01636	1	0.834	+	0.00049	0.00124	0.889	Y	N
Proteasome subunit beta type-4	+	0.00136	0.60958	1.111	+	0.03592	1	1.143	+	0.00066	0.00155	1.213	Y	N
Beta-glucuronidase	+	0.00266	0.83128	0.643	+	0.02986	1	0.630	N/D	N/D	N/D	N/D	N/D	N
Mitotic checkpoint protein BUB3	+	0.00728	1	1.113	+	0.04702	1	1.112	+	0.03503	0.04592	1.057	Y	N
Platelet-activating factor acetylhydrolase IB subunit gamma	+	0.04161	1	1.221	+	0.02117	1	1.266	+	0.01903	0.02649	0.850	N	N
2-hydroxyacyl-CoA lyase 1	+	0.01337	1	1.258	+	0.04677	1	1.314	+	0.00001	0.00010	0.575	N	N

(N/D-not detection, N-no, Y-yes)

**Fig. 2:**
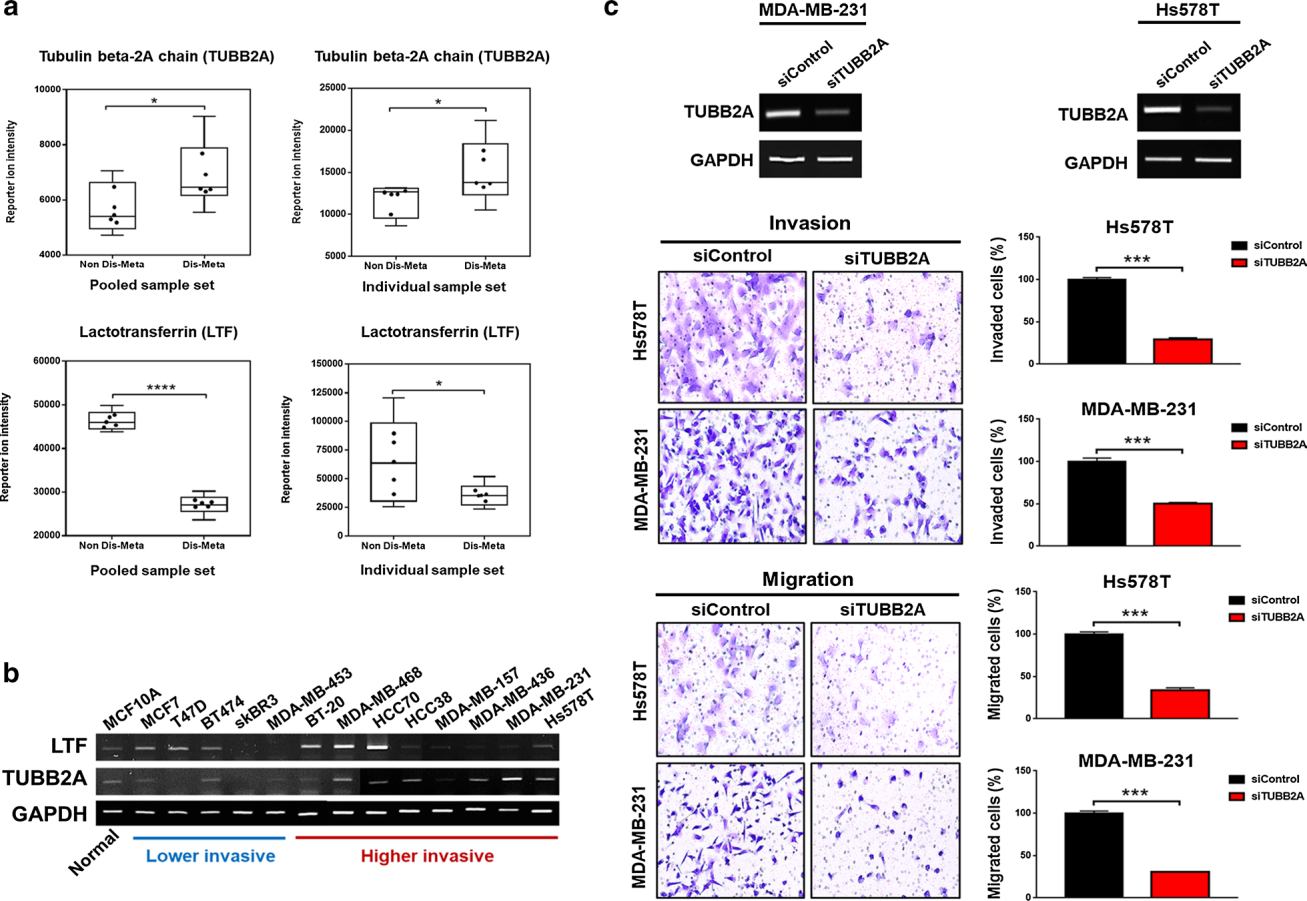
Validation of TUBB2A and LTF as protein targets. **a** Protein expression patterns of TUBB2A and LTF by mass spectrometry; expression pattern of reporter ion intensity of TUBB2A (upper panel) and LTF (lower panel) in pooled sample set (left panel) and individual sample set (right panel), respectively. The data in the interquartile range are displayed as black dots (* < p-value 0.05; **** < p-value 0.0001).**b** Expression patterns of TUBB2A and LTF in various breast cancer cell lines by RT PCR. Higher expression levels are lighter than lower levels (red line; higher invasive BC cell lines, blue line; lower invasive BC cell lines). **c** Results of invasion and migration assays for TUBB2A using Hs578T and MDA-MB-231 BC cell lines. RT-PCR of TUBB2A, downregulated by siRNA transfection in both cell lines (upper panel). Images of invading and migrating cells (lower left panel) and percentage (%) of invading and migrating cells (lower right panel) (*** < p-value 0.001)

Subsequently, we examined whether the overlapping 17 proteins were also differentially expressed in the proteomic dataset of BC cell lines, comparing less-invasive T47D and highly invasive MDA-MB-231 cells. This examination was performed to identify proteins that might have molecular features that are related to the distant metastasis of breast cancer by comparing the BC FFPE and BC cell line proteomes. Five proteins had consistent expression patterns between all proteomic datasets: tubulin beta-2A chain (TUBB2A); lactotransferrin (LTF); acyl-coenzyme a dehydrogenase, C-4 to C-12 straight chain, isoform CRA_a (ACADM); proteasome subunit beta type-4 (PSMB4); and mitotic checkpoint protein BUB3 (BUB3) (Table 1). Next, with regard to the five proteins, the fold-change in expression between nondistant metastatic and distant metastatic groups was calculated. When the fold-change cutoff was set to 1.2, two proteins were selected: LTF was the most extensively downregulated protein, whereas TUBB2A was the most highly upregulated (Fig. 1, Table 1). The normalized abundance of LTF and TUBB2A distinguished the 2 sample groups significantly (Fig. 2a). Based on the criteria, LTF and TUBB2A were selected as important protein targets for validation of their function in relation to distant metastasis of breast cancer.

### Expression levels of TUBB2A and LTF verified by RT-PCR

The difference in the expression of TUBB2A and LTF was validated by RT-PCR in 1 normal breast cell line and 13 breast cancer cell lines, the relative invasiveness of which was determined per other studies [74–81]. The expression of LTF was lower in the higher invasive group than in the lower invasive group, except in 3 cell lines (BT20, MDA-MB-368, and HCC70). In particular, HCC70 expressed the most LTF (Fig. 2b). The level of TUBB2A was generally higher in the higher invasive group compared with the lower invasive group. Specifically, MDA-MB-231 had the highest expression of TUBB2A (Fig. 2b). The expression level of TUBB2A by MS was consistent with that by RT-PCR. The patterns of LTF by MS were not consistent with the RT-PCR results.

### Distant metastatic potential of TUBB2A

The correlation between TUBB2A and metastatic characteristics was validated by invasion and migration assay. Two highly invasive BC cell lines (Hs578T and MDA-MB-231) were used to examine invasion and migration, based on the levels of TUBB2A. As a result, by siRNA transfection, TUBB2A was downregulated in both cell lines by RT-PCR. The number of invading cells fell significantly by over 50% when TUBB2A was knocked down compared with the control group (siControl), as did the number of migrating cells (Fig. 2c). Conversely, because the relative cell proliferation did not differ significantly on the day when the invasion and migration assays were conducted (Additional file 2: Fig. S6), the decreased invasiveness of the cells did not result from the altered cell proliferation. Thus, the distant metastatic potential of TUBB2A was verified, independent of the influence of cell proliferation.

To determine the ability of TUBB2A as a novel protein biomarker candidate of distant metastatic breast cancer, its performance was evaluated in the individual sample set. The sensitivity, specificity, and positive predictive value (PPV) by receiver operating characteristic (ROC) analysis were 78%, 100%, and 88%, respectively. Furthermore, the area under curve (AUC) value was 0.852, based on the ROC curve, and the threshold value, expressed as reporter ion intensity, that corresponded to the highest Youden’s index was 13,178 (Additional file 2: Fig. S7). Based on these results, we expected TUBB2A to perform well in the diagnosis and prediction of distant metastatic breast cancer.

### Biological functions of distant metastatic breast cancer

To examine the functional signatures of distant metastatic breast cancer, we performed a bioinformatics analysis using 259 DEPs from the 2 sample sets. By gene ontology (GO) enrichment analysis, the 177 upregulated proteins in the distant metastasis group were assigned to various biological processes, such as cell–cell adhesion, proteolysis during cellular protein catabolism, NIK/NK-kappa B signaling, microtubule-based processes, and retrograde vesicle-mediated transport,- Golgi-to-ER (Fisher’s exact test p-value < 0.05) (Additional file 2: Fig. S8a, Additional file 5: Table S4). The most significant biological process in upregulated proteins was the regulation of mRNA stability (p-value = 7.82E−07). Conversely, the 82 downregulated proteins were allocated to various biological processes, including oxidation–reduction, organization of actin cytoskeleton, response to hydrogen peroxide, thrombin receptor signaling, sequestering of actin monomers, and positive regulation of toll-like receptor 4 signaling (Fisher’s exact test p-value < 0.05) (Additional file 2: Fig. S8b, Additional file 5: Table S4). The most significant biological process in downregulated proteins was oxidation–reduction (p-value = 2.89E–04).

In the enrichment of biological functions and pathways, the 259 DEPs were assigned to 6 canonical pathways and 11 downstream biological functions (Fisher’s exact test p-value < 0.05, and Z-score > 1). Canonical pathways included acute phase response signaling, ILK signaling, actin cytoskeletal signaling, leukocyte extravasation signaling, and tRNA charging (Additional file 2: Fig. S9a, Additional file 6: Table S5). The most significant and activated canonical pathway was glycolysis I (p-value = 1.74E−06, and activation Z-score = 2.236). Biological functions included polarization of tumor cell lines, orientation of cells, adhesion of BC cell lines, binding of NFkB sites, glycolysis in tumor cell lines, and proliferation of tumor/carcinoma cell lines (Additional file 2: Fig. S9b, Additional file 6: Table S5). The most significant and activated biological function was cell proliferation of tumor cell lines (p-value = 1.69E−08, and activation Z-score = 2.451). Based on our results, we propose that the interaction of various biological functions induces distant metastatic breast cancer.

Of the 2 protein targets, the result showed that the TUBB2A has association with the proliferation of tumor/carcinoma cell lines, microtubule-based processes, epithelial adherens junction signaling, 14-3-3-mediated signaling, and phagosome maturation. The most significant function of TUBB2A was cell proliferation of tumor cell lines (p-value = 1.69E−08). LTF was involved in the binding of NFkB sites, negative regulation of apoptotic process, positive regulation of I-KappaB kinase/NF-kappaB signaling, negative regulation of ATPase activity, and positive regulation of toll-like receptor 4 signaling pathway. Binding of NFkB sites was the most significant function (p-value = 2.17E−04) (Additional file 2: Fig. S10, Additional file 7: Table S6). Thus, these candidates had distinct and independent biological characteristics.

### Proteomic alterations in distant metastatic breast cancer between molecular subtypes

According to the results of a previous study, pooling biological groups can reduce the variation that originates from the sample while retaining the defining features of the group itself [57]. We expected our pooled samples for each molecular subtype to reveal distinct information on the molecular characteristics between the HER2, TNBC, and luminal groups. For these reasons, a pooled sample set was used to identify the changes in proteins between distinct breast cancer molecular subtypes in the distant metastasis and nondistant metastasis groups.

**Fig. 3:**
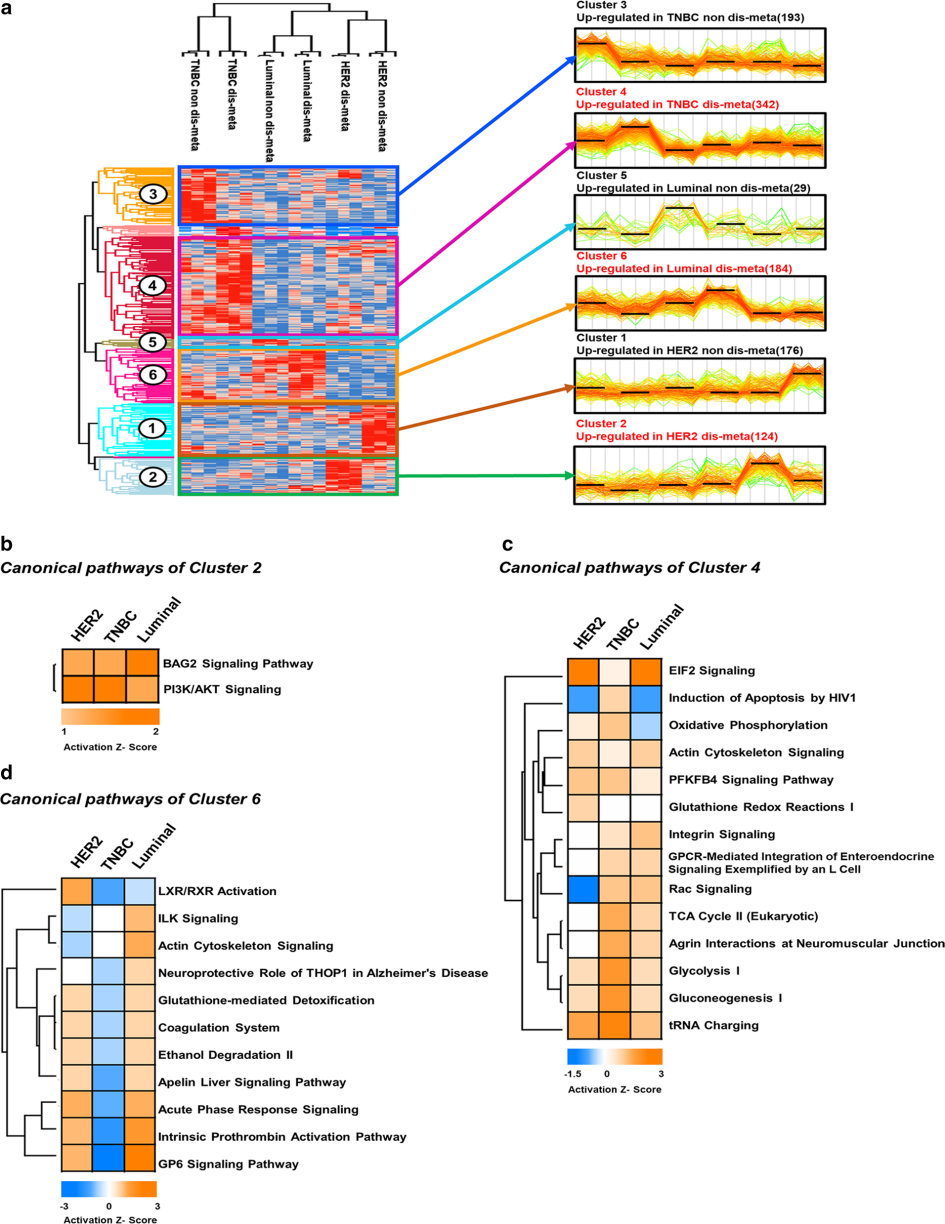
Proteomic alteration in distant metastatic breast cancer between molecular subtypes. **a** Hierarchical clustering of differentially expressed proteins (DEPs) between distant metastatic breast cancer molecular subtypes (ANOVA, p-value < 0.05). The DEPs (1086) from the pooled sample set were divided into 6 groups. Clusters of upregulated proteins are marked in red. **b–d** Canonical pathway enrichment of clusters 2, 4, and 6. The significant pathways (Fisher’s exact test p-value < 0.05) were deduced using Ingenuity Pathway Analysis (IPA), and their activation and inhibition states are expressed as Z-scores

By ANOVA, 1086 proteins were differentially expressed between breast cancer molecular subtypes (p-value < 0.05) (Fig. 3a, Additional file 8: Table S7). These DEPs were then analyzed by hierarchical clustering to determine their expression patterns between breast cancer molecular subtypes, resulting in 6 groups: upregulated proteins in HER2-non-distant metastasis (cluster 1; 176 DEPs), upregulated proteins in HER2-distant metastasis (cluster 2; 124 DEPs), upregulated proteins in TNBC-non-distant metastasis (cluster 3; 193 DEPs), upregulated proteins in TNBC-distant metastasis (cluster 4; 342 DEPs), upregulated proteins in luminal-non-distant metastasis (cluster 5; 29 DEPs), and upregulated proteins in luminal-distant metastasis (cluster 6; 184 DEPs).

### Biological functions of distant metastatic breast cancer between molecular subtypes

To gain greater insight into the molecular features of distant metastatic breast cancer between molecular subtypes, pathway enrichment analysis was conducted for clusters 2, 4, and 6, which comprised proteins that were upregulated in the distant metastasis group of each molecular subtype. By Ingenuity Pathway Analysis (IPA), 2 canonical pathways were derived for cluster 2, versus 14 for cluster 4 and 11 for cluster 6 (p-value < 0.05, Z-score > 1) (Fig. 3b–d, Additional file 9: Table S8). Specifically, in cluster 2, only PI3K/AKT signaling and BAG signaling were deduced and activated between three subtypes. PI3K/AKT signaling was the most highly activated pathway (Z-score = 2) in the HER2 type (Fig. 3b, Additional file 9: Table S8). In cluster 4, all 14 pathways were activated—glycolysis 1, gluconeogenesis 1, and tRNA charging were extensively activated in the TNBC types (Fig. 3c, Additional file 9: Table S8). tRNA charging was the most highly activated pathway (Z-score = 2.828), whereas EIF2 signaling was the least activated (Z-score = 0.333) in TNBC types (Fig. 3c, Additional file 9: Table S8). In cluster 6, most pathways were activated, such as actin cytoskeleton signaling, acute phase response signaling, intrinsic prothrombin activation, and GP6 signaling, in the luminal type. Among them, GP6 signaling was the most highly activated (Z-score = 3.464). However, LXR/RXR signaling was inhibited in the luminal type (Z-score = − 0.707) (Fig. 3d, Additional file 9: Table S8). Based on our results, distinct activation states exist between the HER2, TNBC, and luminal types.

## Discussion

One of the goals of our study was to discover novel protein biomarker candidates of distant metastatic breast cancer. Initially, we considered the potential problem with multiple comparisons, which can generate false positives if unaddressed, in selecting the protein targets. Therefore, we applied a multiple testing correction to our datasets. However, none of proteins was able to pass the BH FDR cutoff. Thus, we proposed alternative criteria to compensate for the statistically insufficient significance of proteins in determining the protein targets.

When the criteria were applied to our in-depth proteome data, LTF (p-value < 0.001) and TUBB2A (p-value < 0.05) appeared as important protein targets for validation of distant metastatic potential. TUBB2A was upregulated and LTF was downregulated in the distant metastasis group. TUBB2A was upregulated in more invasive breast cancer cell lines (i.e., BC cell lines in the higher invasive group), whereas the expression patterns of LTF were perturbed across breast cancer cell lines by RT-PCR. Considering the expression level of TUBB2A in the higher-invasiveness group and the high malignancy of distant metastatic breast cancer [4, 58, 59], the upregulation of TUBB2A might promote the invasion of breast cancer cells, inducing the potential of distant metastatic breast cancer. In addition, based on the results of the invasion and migration assay, we verified that the high expression of TUBB2A increases the mobility of breast cancer cells, providing further support for TUBB2A as a novel biomarker candidate of distant metastatic breast cancer.

Regarding performance of TUBB2A, TUBB2A could distinguish between distant metastasis and nondistant metastasis (i.e., 78% sensitivity, 100% specificity, and an AUC value of 0.852) and might predict distant metastasis (i.e., 88% PPV) in the individual sample set. However, because our TMT-based data were obtained from a small cohort (n = 36), future studies should evaluate the performance of TUBB2A by absolute quantitation in a large cohort to assess its clinical applicability, which lies beyond the scope of our current study. One possible design would be to quantify TUBB2A using targeted proteomic techniques, such as multiple reaction monitoring (MRM) and parallel reaction monitoring (PRM).

Another goal was to determine the overall biological functions that exist in distant metastatic breast cancer. Biological functions that are related to proliferation and movement of cancer cells were activated. Specifically, cell polarization/orientation was related to cell adhesion, and actin-based signaling was associated with migration [60–62]. NF-kappa B modulates the immune response, but its inhibition and dysregulation are linked to improper immune development [63, 64]. Thus, the inhibition of polarization of tumor cell lines and adhesion of BC cell lines might weaken the adhesion between cells in primary breast tumors, and the activation of actin cytoskeletal signaling and proliferation of tumor cell lines might enhance the movement of breast cancer cells. In addition, blocking NF kappa B binding sites might allow breast cancer cells to migrate to other distal sites without activating the immune system.

We noted proteins that were associated with distant metastatic breast cancer, based on our bioinformatics analysis. By GO analysis, ‘cell–cell adhesion’ terms were observed in upregulated and downregulated DEPs. However, each term consisted of different proteins. Furthermore, proteins in ‘adhesion of BC cell lines’ term did not overlap with those in the ‘cell–cell adhesion’ term. Thus, adhesion between breast cancer cells in primary tumors might be weakened, but that between breast cancer cells and cells in other organs could be strengthened, due to various proteins with potentially distinct functions in cell adhesion. In our pathway enrichment analysis, FN1 overlapped between activated leukocyte extravasation signaling and inhibited acute phase response signaling. Considering the opposing states of these pathways, the former might enhance the mobility of breast cancer cells to other organs, shuttling leukocytes out of the circulatory system. In parallel, inhibition of acute phase response signaling might suppress the immune response. Thus, FN1 might create a suitable microenvironment that is conducive to distant metastasis of breast cancer.

With regard to our protein targets, TUBB2A was associated with cellular proliferation, movement, and adhesion, and LTF was involved in cell death, the immune response, and metabolism. Based on these functions, TUBB2A might control the mobility of distant metastatic breast cancer by regulating the adhesion and proliferation of breast cancer cells, and LTF might govern the death of breast cancer cells and the immune system during distant metastasis. Thus, TUBB2A might be a key protein that controls the migration of breast cancer cells from a primary tumor. LTF might be an auxiliary protein that helps breast cancer cells survive during movement toward distal sites by disrupting the immune system.

Another goal was to determine the characteristics of distant metastatic breast cancer between molecular subtypes. In cluster 2, the most highly activated pathway was PI3K/AKT signaling in the HER2 type. A previous study that used transcriptome data revealed that PI3K/AKT kinases are expressed in circulating breast tumor cells and that the activation of this signal regulates their metastatic and malignant state [68]. Compared with our proteomic results, the activation states of PI3K/AKT signaling were consistent. Thus, our PI3K/AKT signaling proteins might be associated with the regulation of distant metastatic potential and function as targets for the eradication of HER2-type distant metastatic breast cancer.

In cluster 4, the most highly activated pathway was tRNA charging signaling in the TNBC type. The exact functions of this pathway in distant metastatic breast cancer have not been determined. However, based on a previous study, tRNA overexpression in breast tumor cells might increase the translational efficiency of genes that are related to the progression and development of breast cancer [67]. The tRNA charging-related proteins that we recorded might be upregulated and translationally modified products of such genes, influencing the distant metastatic potential and progression of breast cancer. Thus, these proteins might be targets for removal or suppression in slowing the malignancy of TNBC-type distant metastatic breast cancer.

In cluster 6, the most highly activated pathway was glycoprotein 6 (GP6) signaling in the luminal type. GP6 is a platelet membrane glycoprotein that functions as a receptor for collagen and regulates the collagen-induced activation and aggregation of platelets [65, 66]. The detailed functions of this pathway in distant metastatic breast cancer have not been described. However, based on its functions, breast cancer cells could migrate easily to distal sites, masking their aggregate forms with platelet-combined forms. Furthermore, breast cancer cell complexes might adhere to collagen and subsequently to platelets, leading to additional platelet aggregation. Thus, GP6 signaling and its factors might facilitate the circulation of breast cancer cells with little activation of the immune systems due to their disguised forms, allowing them to settle at distal sites. Furthermore, the expression level of these proteins could be used to monitor the progression of luminal-type distant metastatic breast cancer.

Although we performed pathway enrichment analysis using the upregulated DEPs in the 3 clusters, one of the benefits of our study was that it could have considered the downregulated DEPs in the remaining 3 clusters (clusters 1, 3, and 5) in the analysis. These proteins might be related to distinct biological activities that suppress the activation of distant metastatic breast cancer between subtypes. Consequently, our proteomic clusters might expand our understanding of the effects of molecular subtype on distant metastatic breast cancer.

Without our in-depth proteomic data, most of our DEPs might be unable to be identified or detected in other studies, because we are the first to collect proteomic data in distant metastatic breast cancer, analyzing clinical FFPE tissues from primary breast tumors. Our results indicate that the pathological relevance of our FFPE tissues in BC research is valid at the proteomic level and in severe breast cancer pathologies. Through our latent data, we discovered a novel protein biomarker candidate that has the potential to distinguish distant metastatic breast cancer and demonstrated distinct molecular features between BC subtypes. We expect that our biomarker candidate can be used to diagnose and predict distant metastatic breast cancer. Furthermore, our molecular pathways should provide insights into the relationship between molecular subtypes and distant metastatic breast cancer.

## Conclusions

We have constructed a comprehensive proteome of distant metastatic breast cancer by analyzing FFPE tissue slides using TMT-based mass spectrometric techniques. Our study demonstrates that the TMT-based approach is beneficial, because its greater quantitative ability generates a larger selection of proteins from which to choose novel biomarker candidates. This finding was verified by our proteomic dataset, which comprised the largest number of proteins in distant metastatic breast cancer. Through our criteria, we selected 2 important protein targets for distant metastatic breast cancer and performed functional studies to validate them. Finally, we were able to propose a novel protein biomarker candidate. Furthermore, our bioinformatics analysis revealed specific molecular characteristics between molecular subtypes. Thus, our in-depth proteomic data and analyses can be an important resource for distant metastatic breast cancer research. In future studies, we hope to assemble a larger cohort of breast cancer FFPE samples to test the performance of our novel biomarker candidate using targeted proteomics techniques, such as parallel reaction monitoring (PRM) and multiple reaction monitoring (MRM).

## Supplementary information

**Additional file 1: Table S1.** Clinical information on patients. Clinical information on all 36 patients is listed.

**Additional file 2: Figure S1.** Detailed experimental workflow of TMT-based proteomic study. Graphical representation of the workflow for our TMT experiments. Three sample sets were analyzed using our TMT-based proteomic techniques. **Figure S2.** Identified and quantified proteins in TMT experiments. (a) The number of identified and quantified proteins in the pooled sample set, individual sample set, and cell line set. (B) The number of identified proteins in each sample of the individual sample set. (C) The number of identified proteins in each sample of the pooled sample set. **Figure S3.** Dynamic ranges of protein abundance in pooled sample set and individual sample set. The dynamic range of the pooled sample set is marked in yellow, and that of the individual sample set is marked in blue. Known metastatic biomarkers are indicated in red, and breast cancer markers are marked in black. **Figure S4.** Comparative analysis between our FFPE tissue proteome and those of our previous studies. (a) Comparison of identified proteins between our pooled sample proteome data and those of *MS Jin* et al. (b) Comparison of identified proteins between our individual sample proteome data and those of *MS Jin* et al. **Figure S5.** Quality assessment of MS analysis. (a) Abundance and technical variation of the external standard, ovalbumin. Ovalbumin was quantified in the middle-high abundance interval and had a CV of 4.2% and 6.7% in the pooled and individual sample sets in 18 TMT channels, respectively. (b), (c) The quantitative reproducibility of all proteins was improved slightly on normalization with the external standard, ovalbumin; the median CV value of the biological replicates of the pooled and individual sample sets decreased by 0.36% and 1.54%, respectively. (d) Cross-correlation analysis using the protein levels to confirm the repeatability of the MS analyses between experimental sets of the pooled sample set. (e) Variabilities in individual samples in our MS analysis are depicted in a multiscatter plot. Reproducibility between individual samples is represented by Pearson’s correlation value. Values of correlation with HER2 ND-2 are marked in red. (ND; non dis-meta, D; dis-meta, LU; luminal, - #; number of TMT set). **Figure S6.** Cell proliferation of MDA-MB 231 and Hs578T cell lines. Relative cell proliferation was observed for 3 days, when TUBB2A was knocked down, compared with the control group (siControl) (* < p-value 0.05; ** < p-value 0.01). The time point at which the migration and invasion assays were performed is indicated in the blue circle. **Figure S7.** Performance of the novel biomarker TUBB2A in the individual sample set. Table of summary statistics in ROC analysis, ROC curve with AUC = 0.852, and interactive dot diagram with sensitivity = 78%, specificity = 100%, and reporter ion intensity threshold = 13,178. **Figure S8.** Gene ontology analysis of all 259 DEPs in the two sample sets using The Database for Annotation, Visualization and Integrated Discovery (DAVID). (a) Biological process terms of 177 upregulated DEPs. (b) Biological process terms of 82 downregulated DEPs (Fisher’s exact test p-value < 0.05). **Figure S9.** IPA analysis of all 259 DEPs in the two sample sets regarding canonical pathway, and downstream biological functions. (a) Canonical pathway enrichment of all 259 DEPs in the two sample sets. (b) Hierarchical clustering of downstream biological functions assessed by IPA using the 259 DEPs. The significant pathways, and downstream biological functions (Fisher’s exact test p-value < 0.05) were deduced using Ingenuity Pathway Analysis (IPA), and their activation and inhibition states are expressed as Z-scores. **Figure S10.** Biological functions and canonical pathways related to the two protein targets by IPA and DAVID analysis. Biological functions and pathways of TUBB2A (upper panel) and LTF (lower panel) (Fisher’s exact test p-value < 0.05 for DAVID and IPA analysis).

**Additional file 3: Table S2.** List of all identified proteins in this study. MS information on identified proteins is listed in the pooled and individual sample sets. Normalized protein levels of each sample were used for further statistical analysis.

**Additional file 4: Table S3.** List of significantly differentially expressed proteins (DEPs) by student t-test. Statistically significant DEPs by student’s t-test (p-value < 0.05), fold-changes, p-values, and adjusted p-values (Benjamini–Hochberg FDR cutoff of 0.05) in the pooled sample set, individual sample set, and cell line set. These proteins were used to select protein targets for validation of distant metastatic potential and perform the bioinformatics analysis.

**Additional file 5: Table S4.** GO analysis using the DAVID bioinformatics tool. Biological processes of upregulated and downregulated DEPs by student’s t-test are listed. The p-value (modified Fisher exact p-value) cutoff for the GO annotation was set to < 0.05. Genes that were associated with each GO term are represented as official gene symbols. ‘GO direct’ filters extensive GO terms, based on the measured specificity of each term.

**Additional file 6: Table S5.** Downstream biological functions and canonical pathways of DEPs by student t-test by IPA analysis. Downstream biological functions and canonical pathways were examined using the IPA informatics tool. The p-value cutoff was set to < 0.05, and the activation Z-score was set to > 1. Proteins in each biological function and pathway are listed. P-values and Z-scores of biological functions and pathways are shown.

**Additional file 7: Table S6.** Biological functions of TUBB2A and LTF. Biological functions of TUBB2A and LTF were examined using the IPA and DAVID bioinformatics tools. Biological processes and canonical pathways of TUBB2A and LTF are listed. The p-value cutoff was set to < 0.05 for the IPA analysis. The p-value (modified Fisher exact p-value) cutoff for the GO annotation was set to < 0.05. Proteins in each biological function are listed.

**Additional file 8: Table S7.** List of significantly differentially expressed proteins (DEPs) by ANOVA. Statistical significance and p-values for DEPs by ANOVA (p-value < 0.05) in the pooled and individual sample sets. Significantly differentially expressed proteins in six clusters in each sample set are listed. The proteins in clusters 2, 4, and 6 of the pooled sample set were used to perform a bioinformatics analysis of the molecular characteristics of distant metastatic breast cancer between molecular subtypes. Adjusted p-values for DEPs in the pooled sample set are listed (Benjamini-Hochberg FDR cutoff of 0.05).

**Additional file 9: Table S8.** Canonical pathways of clusters enriched by IPA analysis. Canonical pathways in clusters 2, 4, and 6 of the pooled sample set were investigated using the IPA informatics tool. Canonical pathways between molecular subtypes are listed. The p-value cutoff was set to < 0.05, and the activation Z-score was set to > 1. P-values and Z-scores of the canonical pathways are listed.

## Data Availability

All datasets that were generated and analyzed during this study are included in this published article and its additional information files. The MS proteomic data in this study have been deposited into ProteomeXchange (http://proteomecentral.proteomexchange.org) through the PRIDE partner repository [43]: dataset identifier PXD016061. The datasets are available from the corresponding author on reasonable request. Username: reviewer41229@ebi.ac.uk, Password: 4ybAzlVM.

## Abbreviations

ACN: Acetonitrile
TUBB2A: Tubulin beta 2A chain
LTF: Lactotransferrin
HSPA9: Stress-70 protein, mitochondrial
PSMB4: Proteasome subunit beta type-4
CTNNA1: Catenin alpha-1
XPO5: Exportin-5
PAFAH1B3: Platelet-activating factor acetylhydrolase IB subunit gamma
TMSB10: Thymosin beta-10
TMSB4X: Thymosin beta-4
RAC1: Ras-related protein-Rac1
PFKM: ATP-dependent 6-phosphofructokinase
PKM: Pyruvate kinase PKM
FN1: Fibronectin
GP6: Glycoprotein 6
CV: Coefficient of variation
FDR: False discovery rate
DEP: Differentially expressed protein
FFPE: Formalin-fixed paraffin-embedded
MS: Mass spectrometry
TMT: Tandem mass tag
RT-PCR: Real-time polymerase chain reaction
Dis-meta: Distant metastasis
Non dis-meta: Non-distant metastasis
GO: Gene ontology
HCD: Higher-energy collisional dissociation
AGC: Automatic gain control
NCE: Normalized collision energy
DDA: Data-dependent acquisition
Maximum IT: Maximum ion injection time
HPRP: High-pH reversed-phase
RP: Reverse-phase
FFPE: Formalin-fixed paraffin-embedded
TMT: Tandem mass tag
BC: Breast cancer
MRM: Multiple reaction monitoring
PRM: Parallel reaction monitoring
BH-FDR: Benjamini–Hochberg false discovery rate
