# Supplementary material for: Identification of TUBB2A by quantitative proteomic analysis as a novel biomarker for the prediction of distant metastatic breast cancer
Source: Clin Proteomics. 2020 May 24;17:16. doi: 10.1186/s12014-020-09280-z (PMC7247212; doi:10.1186/s12014-020-09280-z)
Supplement: Supplementary file 2 — Additional file 2: Figure S1. Detailed experimental workflow of TMT-based proteomic study. Graphical representation of the workflow for our TMT experiments. Three sample sets were analyzed using our TMT-based proteomic techniques. Figure S2. Identified and quantified proteins in TMT experiments. (a) The number of identified and quantified proteins in the pooled sample set, individual sample set, and cell line set. (B) The number of identified proteins in each sample of the individual sample set. (C) The number of identified proteins in each sample of the pooled sample set. Figure S3. Dynamic ranges of protein abundance in pooled sample set and individual sample set. The dynamic range of the pooled sample set is marked in yellow, and that of the individual sample set is marked in blue. Known metastatic biomarkers are indicated in red, and breast cancer markers are marked in black. Figure S4. Comparative analysis between our FFPE tissue proteome and those of our previous studies. (a) Comparison of identified proteins between our pooled sample proteome data and those of MS Jin et al. (b) Comparison of identified proteins between our individual sample proteome data and those of MS Jin et al. Figure S5. Quality assessment of MS analysis. (a) Abundance and technical variation of the external standard, ovalbumin. Ovalbumin was quantified in the middle-high abundance interval and had a CV of 4.2% and 6.7% in the pooled and individual sample sets in 18 TMT channels, respectively. (b), (c) The quantitative reproducibility of all proteins was improved slightly on normalization with the external standard, ovalbumin; the median CV value of the biological replicates of the pooled and individual sample sets decreased by 0.36% and 1.54%, respectively. (d) Cross-correlation analysis using the protein levels to confirm the repeatability of the MS analyses between experimental sets of the pooled sample set. (e) Variabilities in individual samples in our MS analysis are depicted in a m [file 12014_2020_9280_MOESM2_ESM.pptx]

## Slide 1
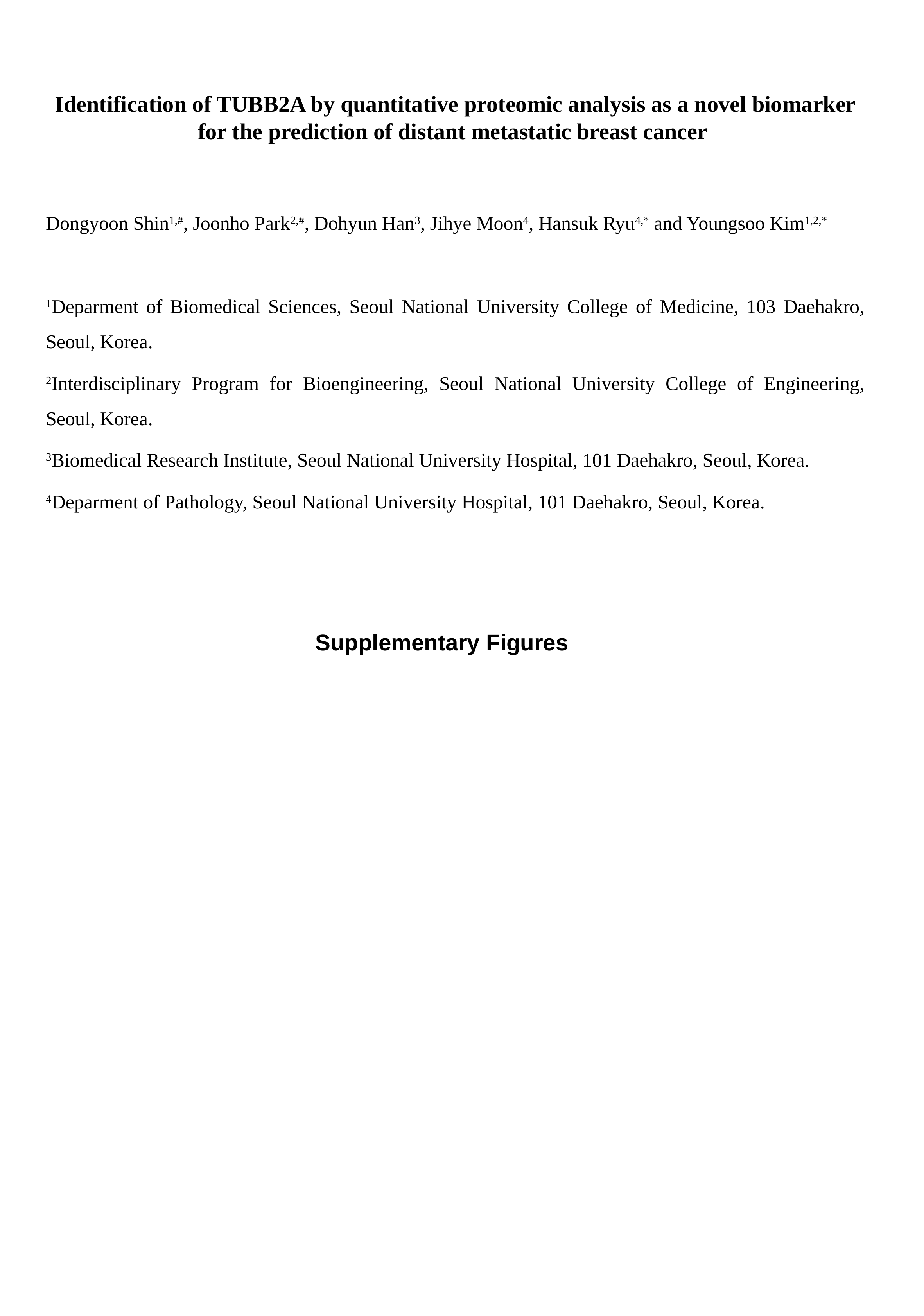

Identification of TUBB2A by quantitative proteomic analysis as a novel biomarker for the prediction of distant metastatic breast cancer
Dongyoon Shin1,#, Joonho Park2,#, Dohyun Han3, Jihye Moon4, Hansuk Ryu4,* and Youngsoo Kim1,2,*
1Deparment of Biomedical Sciences, Seoul National University College of Medicine, 103 Daehakro, Seoul, Korea.
2Interdisciplinary Program for Bioengineering, Seoul National University College of Engineering, Seoul, Korea.
3Biomedical Research Institute, Seoul National University Hospital, 101 Daehakro, Seoul, Korea.
4Deparment of Pathology, Seoul National University Hospital, 101 Daehakro, Seoul, Korea.
Supplementary Figures

## Slide 2
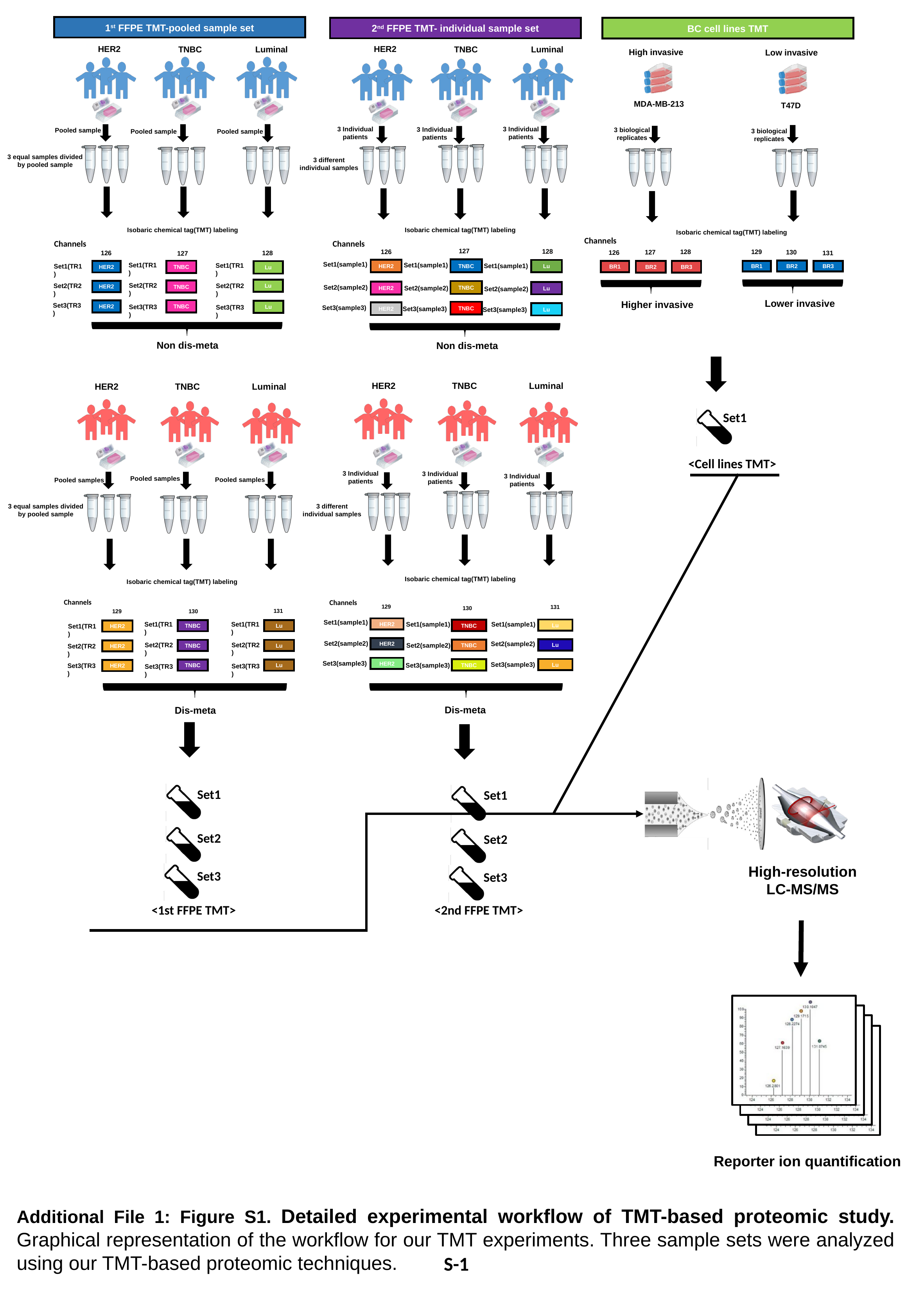

1st FFPE TMT-pooled sample set
2nd FFPE TMT- individual sample set
BC cell lines TMT
HER2
HER2
TNBC
Luminal
TNBC
Luminal
High invasive
Low invasive
MDA-MB-213
T47D
3 Individual
patients
3 Individual
patients
3 Individual
patients
3 biological replicates
Pooled sample
3 biological replicates
Pooled sample
Pooled sample
3 equal samples divided by pooled sample
3 different individual samples
Isobaric chemical tag(TMT) labeling
Isobaric chemical tag(TMT) labeling
Isobaric chemical tag(TMT) labeling
Channels
Channels
Channels
127
128
126
128
129
127
130
126
128
126
131
127
Set1(sample1)
Set1(sample1)
Set1(TR1)
Set1(TR1)
Set1(sample1)
Set1(TR1)
TNBC
HER2
Lu
BR2
TNBC
BR1
BR3
BR2
BR3
BR1
HER2
Lu
Set2(TR2)
Set2(TR2)
Set2(TR2)
Lu
Set2(sample2)
TNBC
HER2
TNBC
Set2(sample2)
HER2
Lu
Set2(sample2)
Lower invasive
Higher invasive
Set3(TR3)
Set3(TR3)
TNBC
Set3(TR3)
HER2
Lu
Set3(sample3)
TNBC
Set3(sample3)
HER2
Set3(sample3)
Lu
Non dis-meta
Non dis-meta
HER2
TNBC
Luminal
HER2
TNBC
Luminal
Set1
<Cell lines TMT>
3 Individual
patients
3 Individual
patients
3 Individual
patients
Pooled samples
Pooled samples
Pooled samples
3 equal samples divided by pooled sample
3 different individual samples
Isobaric chemical tag(TMT) labeling
Isobaric chemical tag(TMT) labeling
Channels
Channels
129
131
130
131
129
130
Set1(sample1)
Set1(sample1)
Set1(sample1)
Set1(TR1)
Set1(TR1)
HER2
Set1(TR1)
Lu
TNBC
TNBC
Lu
HER2
Set2(sample2)
Set2(sample2)
Set2(TR2)
Set2(TR2)
HER2
Set2(sample2)
Set2(TR2)
Lu
TNBC
TNBC
Lu
HER2
Set3(sample3)
Set3(sample3)
HER2
Set3(sample3)
Set3(TR3)
Lu
TNBC
Set3(TR3)
Set3(TR3)
TNBC
Lu
HER2
Dis-meta
Dis-meta
Set1
Set1
Set2
Set2
High-resolution
LC-MS/MS
Set3
Set3
<1st FFPE TMT>
<2nd FFPE TMT>
Reporter ion quantification
Additional File 1: Figure S1. Detailed experimental workflow of TMT-based proteomic study. Graphical representation of the workflow for our TMT experiments. Three sample sets were analyzed using our TMT-based proteomic techniques.
S-1

## Slide 3
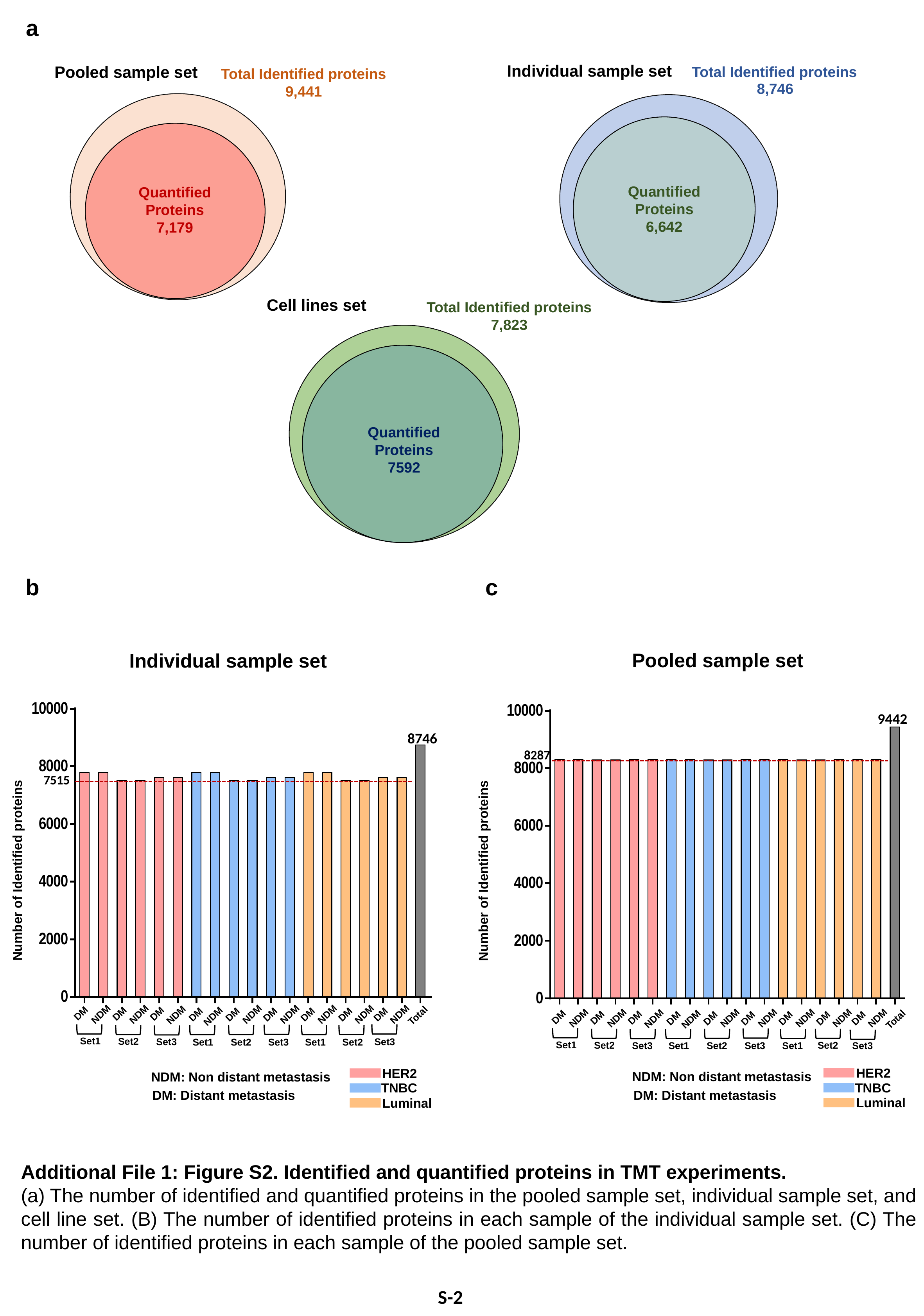

a
Individual sample set
Total Identified proteins
8,746
Quantified
Proteins
6,642
Pooled sample set
Total Identified proteins
9,441
Quantified
Proteins
7,179
Cell lines set
Total Identified proteins
7,823
Quantified
Proteins
7592
c
b
Pooled sample set
Individual sample set
9442
8746
8287
7515
Number of Identified proteins
Number of Identified proteins
DM
DM
DM
NDM
NDM
DM
DM
NDM
DM
NDM
DM
NDM
DM
NDM
DM
NDM
NDM
Total
NDM
DM
DM
DM
NDM
NDM
DM
DM
NDM
DM
NDM
DM
NDM
DM
NDM
DM
NDM
NDM
NDM
Total
Set1
Set2
Set3
Set3
Set1
Set2
Set3
Set1
Set2
Set1
Set2
Set2
Set3
Set3
Set1
Set2
Set3
Set1
HER2
HER2
NDM: Non distant metastasis
NDM: Non distant metastasis
TNBC
TNBC
DM: Distant metastasis
DM: Distant metastasis
Luminal
Luminal
Additional File 1: Figure S2. Identified and quantified proteins in TMT experiments.
(a) The number of identified and quantified proteins in the pooled sample set, individual sample set, and cell line set. (B) The number of identified proteins in each sample of the individual sample set. (C) The number of identified proteins in each sample of the pooled sample set.
S-2

## Slide 4
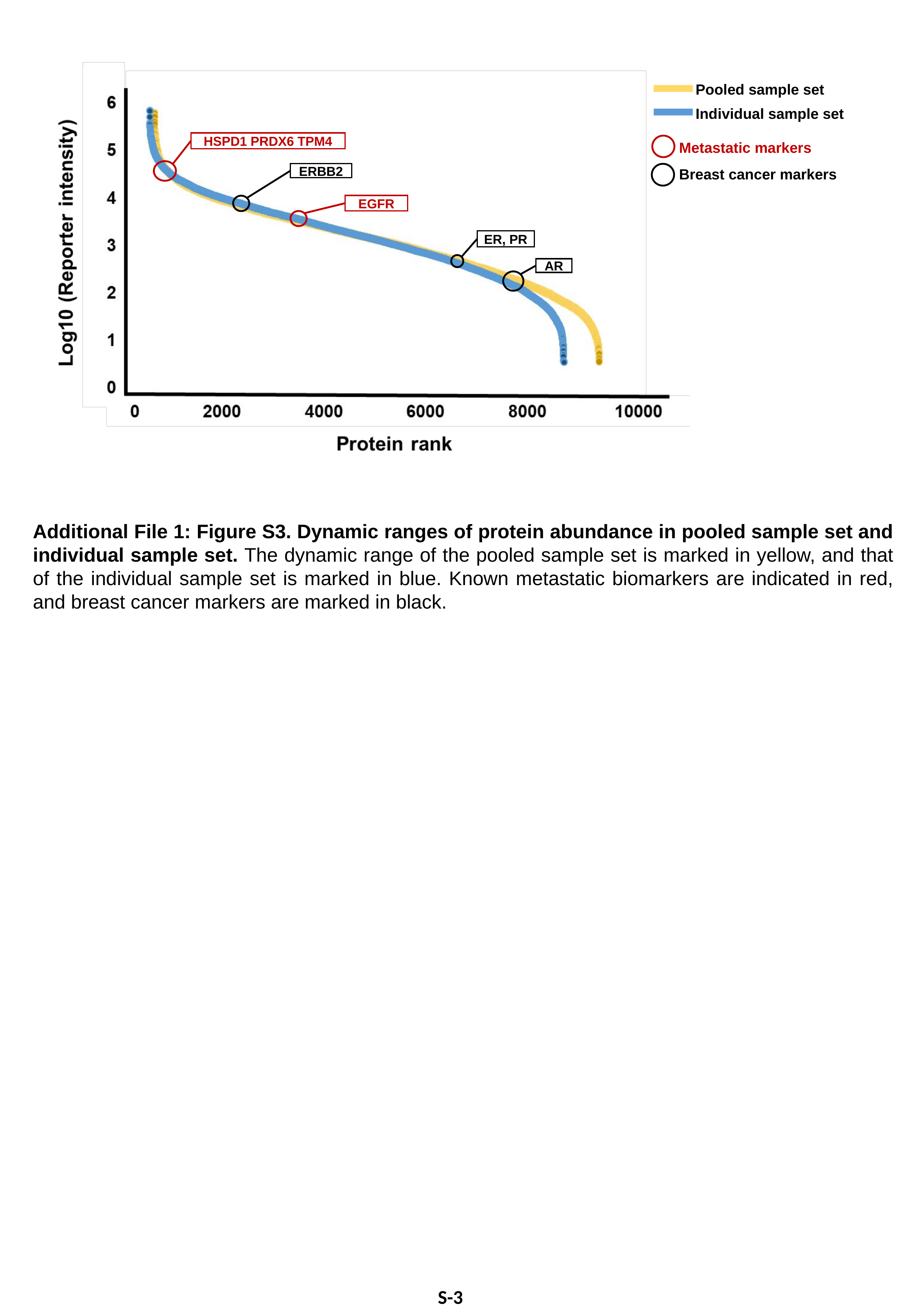

Pooled sample set
Individual sample set
HSPD1 PRDX6 TPM4
Metastatic markers
Breast cancer markers
ERBB2
EGFR
ER, PR
AR
Additional File 1: Figure S3. Dynamic ranges of protein abundance in pooled sample set and individual sample set. The dynamic range of the pooled sample set is marked in yellow, and that of the individual sample set is marked in blue. Known metastatic biomarkers are indicated in red, and breast cancer markers are marked in black.
S-3

## Slide 5
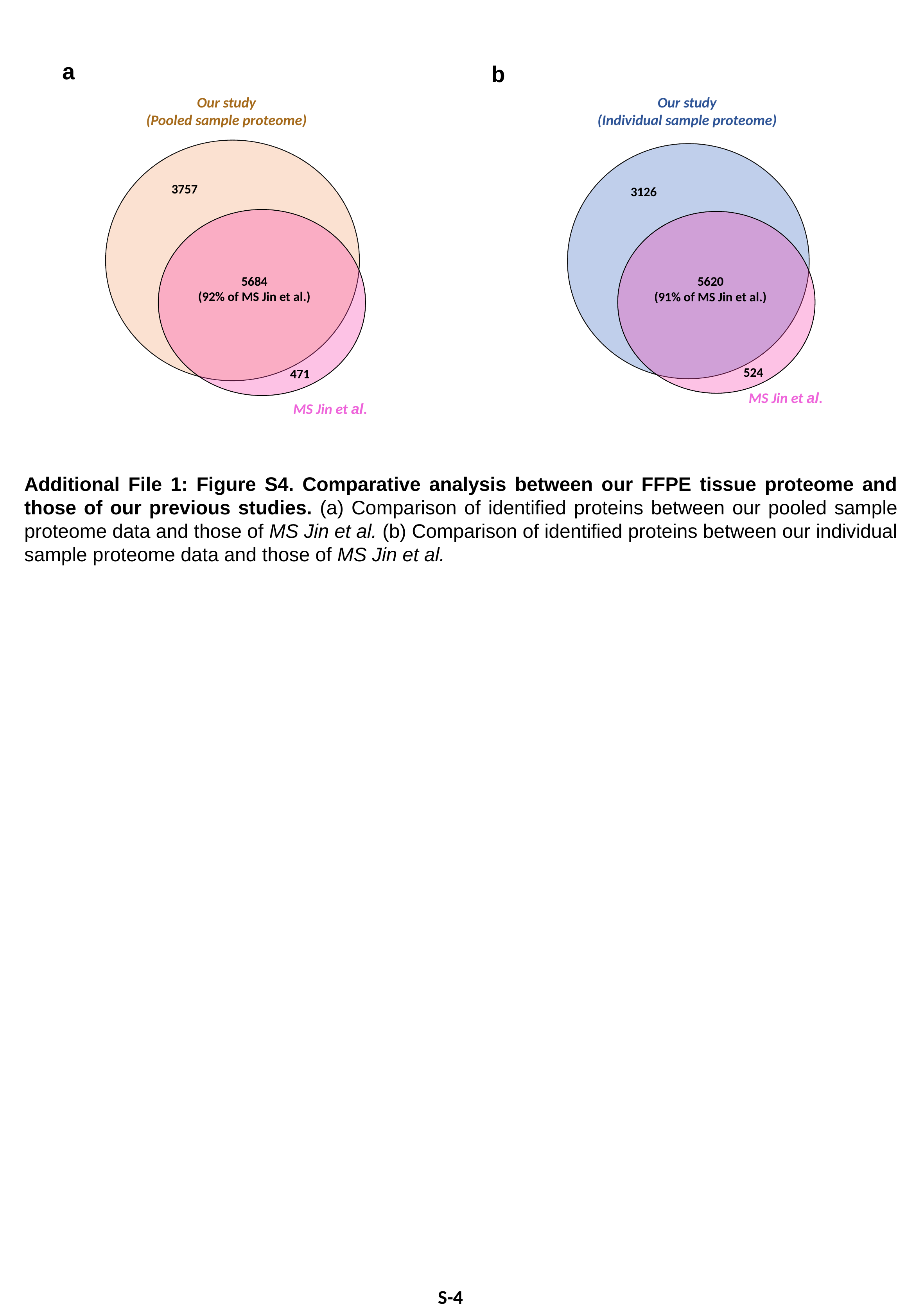

a
b
Our study
(Pooled sample proteome)
Our study
(Individual sample proteome)
3757
3126
5684
(92% of MS Jin et al.)
5620
(91% of MS Jin et al.)
524
471
MS Jin et al.
MS Jin et al.
Additional File 1: Figure S4. Comparative analysis between our FFPE tissue proteome and those of our previous studies. (a) Comparison of identified proteins between our pooled sample proteome data and those of MS Jin et al. (b) Comparison of identified proteins between our individual sample proteome data and those of MS Jin et al.
S-4

## Slide 6
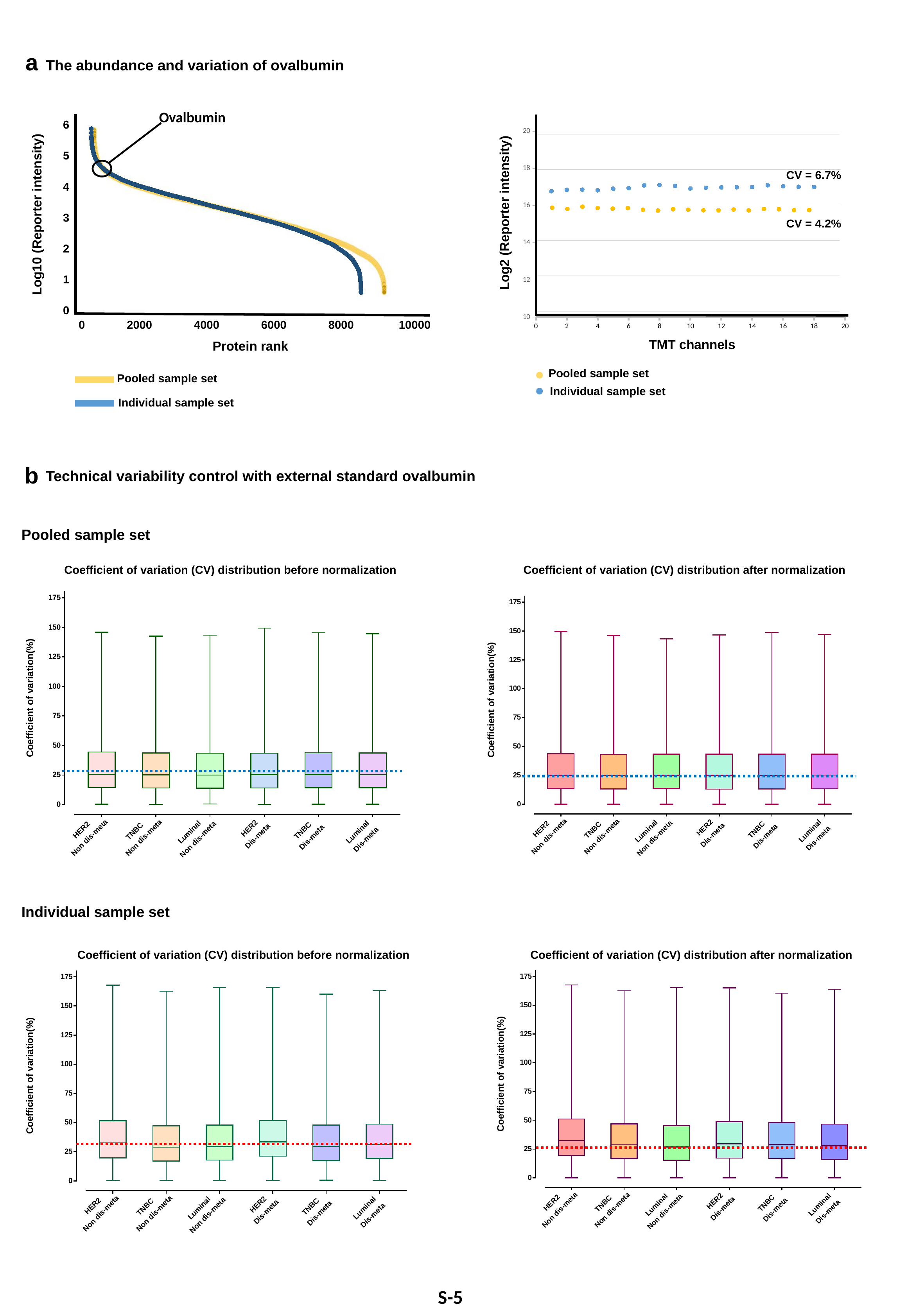

a
The abundance and variation of ovalbumin
### Chart
| Category | |
|---|---|
6
5
4
3
2
1
0
### Chart
| Category | |
|---|---|
Ovalbumin
### Chart
| Category | |
|---|---|
CV = 6.7%
Log2 (Reporter intensity)
Log10 (Reporter intensity)
CV = 4.2%
 0 2000 4000 6000 8000 10000
TMT channels
Protein rank
Pooled sample set
Pooled sample set
Individual sample set
Individual sample set
b
Technical variability control with external standard ovalbumin
Pooled sample set
Coefficient of variation (CV) distribution after normalization
Coefficient of variation (CV) distribution before normalization
Individual sample set
Coefficient of variation (CV) distribution after normalization
Coefficient of variation (CV) distribution before normalization
S-5

## Slide 7
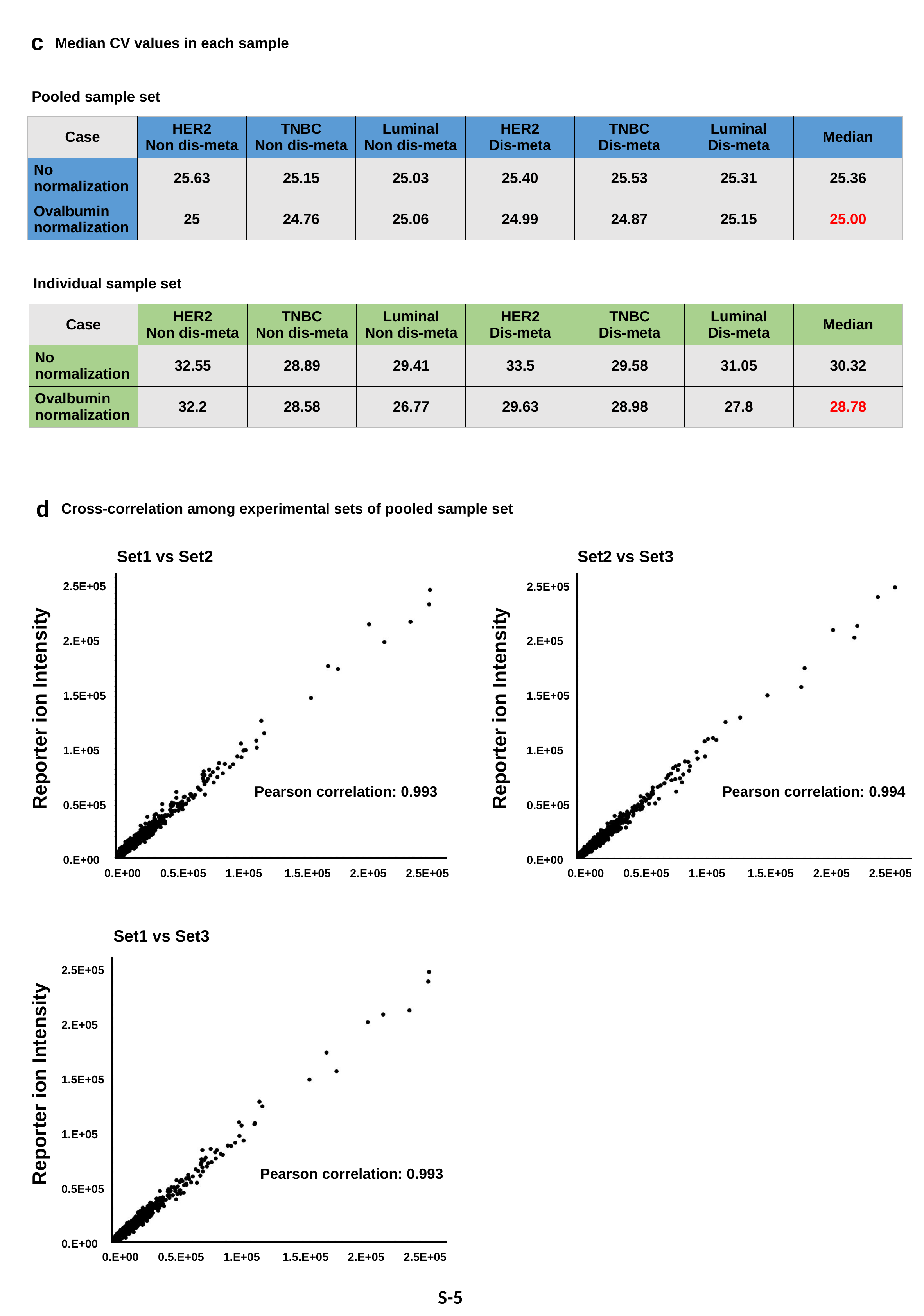

c
Median CV values in each sample
Pooled sample set
| Case | HER2 Non dis-meta | TNBC Non dis-meta | Luminal Non dis-meta | HER2 Dis-meta | TNBC Dis-meta | Luminal Dis-meta | Median |
| --- | --- | --- | --- | --- | --- | --- | --- |
| No normalization | 25.63 | 25.15 | 25.03 | 25.40 | 25.53 | 25.31 | 25.36 |
| Ovalbumin normalization | 25 | 24.76 | 25.06 | 24.99 | 24.87 | 25.15 | 25.00 |
Individual sample set
| Case | HER2 Non dis-meta | TNBC Non dis-meta | Luminal Non dis-meta | HER2 Dis-meta | TNBC Dis-meta | Luminal Dis-meta | Median |
| --- | --- | --- | --- | --- | --- | --- | --- |
| No normalization | 32.55 | 28.89 | 29.41 | 33.5 | 29.58 | 31.05 | 30.32 |
| Ovalbumin normalization | 32.2 | 28.58 | 26.77 | 29.63 | 28.98 | 27.8 | 28.78 |
d
Cross-correlation among experimental sets of pooled sample set
Set1 vs Set2
Set2 vs Set3
2.5E+05
2.E+05
1.5E+05
1.E+05
0.5E+05
0.E+00
2.5E+05
2.E+05
1.5E+05
1.E+05
0.5E+05
0.E+00
Reporter ion Intensity
Reporter ion Intensity
Pearson correlation: 0.993
Pearson correlation: 0.994
0.E+00 0.5.E+05 1.E+05 1.5.E+05 2.E+05 2.5E+05
0.E+00 0.5.E+05 1.E+05 1.5.E+05 2.E+05 2.5E+05
Set1 vs Set3
2.5E+05
2.E+05
1.5E+05
1.E+05
0.5E+05
0.E+00
Reporter ion Intensity
Pearson correlation: 0.993
0.E+00 0.5.E+05 1.E+05 1.5.E+05 2.E+05 2.5E+05
S-5

## Slide 8
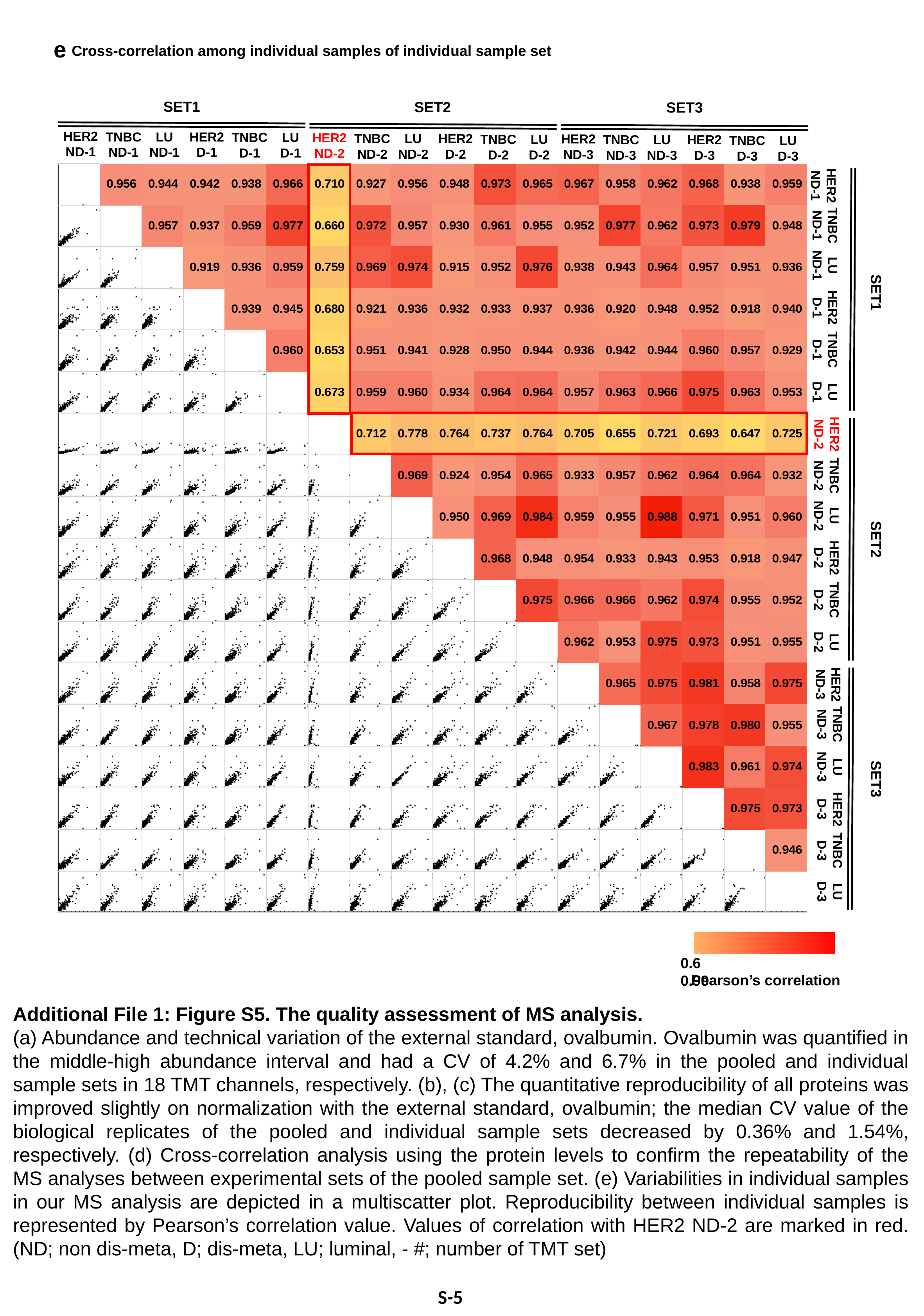

e
Cross-correlation among individual samples of individual sample set
SET1
SET2
SET3
HER2
ND-1
TNBCND-1
LU
ND-1
HER2
D-1
TNBCD-1
LU
D-1
HER2
ND-2
TNBCND-2
LU
ND-2
HER2
D-2
HER2
ND-3
TNBCD-2
LU
D-2
TNBCND-3
LU
ND-3
HER2
D-3
TNBCD-3
LU
D-3
HER2
ND-1
TNBCND-1
LU
ND-1
SET1
HER2
D-1
TNBCD-1
LU
D-1
HER2
ND-2
TNBCND-2
LU
ND-2
SET2
HER2
D-2
TNBCD-2
LU
D-2
HER2
ND-3
TNBCND-3
LU
ND-3
SET3
HER2
D-3
TNBCD-3
LU
D-3
0.6 0.99
Pearson’s correlation
Additional File 1: Figure S5. The quality assessment of MS analysis.
(a) Abundance and technical variation of the external standard, ovalbumin. Ovalbumin was quantified in the middle-high abundance interval and had a CV of 4.2% and 6.7% in the pooled and individual sample sets in 18 TMT channels, respectively. (b), (c) The quantitative reproducibility of all proteins was improved slightly on normalization with the external standard, ovalbumin; the median CV value of the biological replicates of the pooled and individual sample sets decreased by 0.36% and 1.54%, respectively. (d) Cross-correlation analysis using the protein levels to confirm the repeatability of the MS analyses between experimental sets of the pooled sample set. (e) Variabilities in individual samples in our MS analysis are depicted in a multiscatter plot. Reproducibility between individual samples is represented by Pearson’s correlation value. Values of correlation with HER2 ND-2 are marked in red. (ND; non dis-meta, D; dis-meta, LU; luminal, - #; number of TMT set)
S-5

## Slide 9
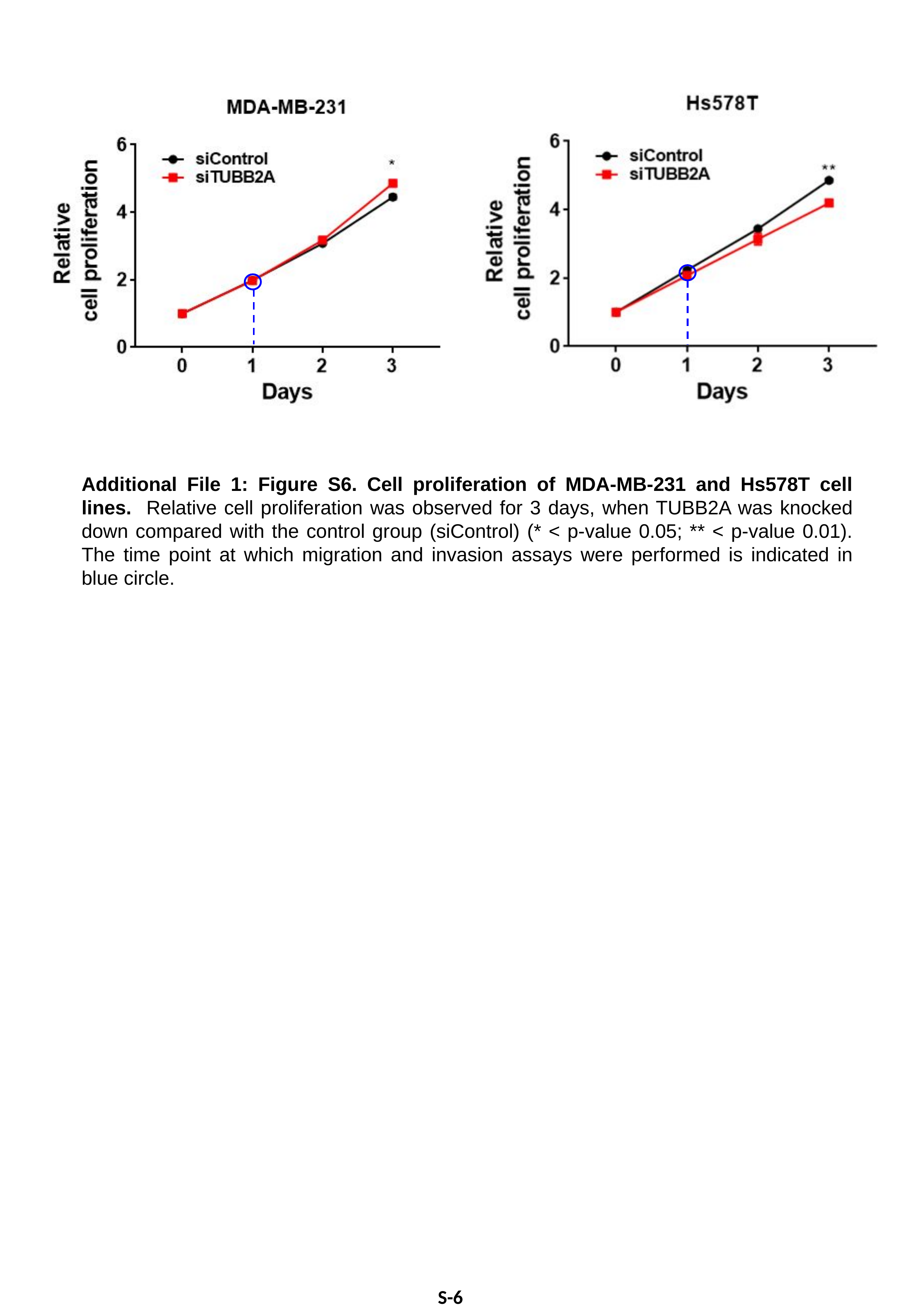

Additional File 1: Figure S6. Cell proliferation of MDA-MB-231 and Hs578T cell lines. Relative cell proliferation was observed for 3 days, when TUBB2A was knocked down compared with the control group (siControl) (* < p-value 0.05; ** < p-value 0.01). The time point at which migration and invasion assays were performed is indicated in blue circle.
S-6

## Slide 10
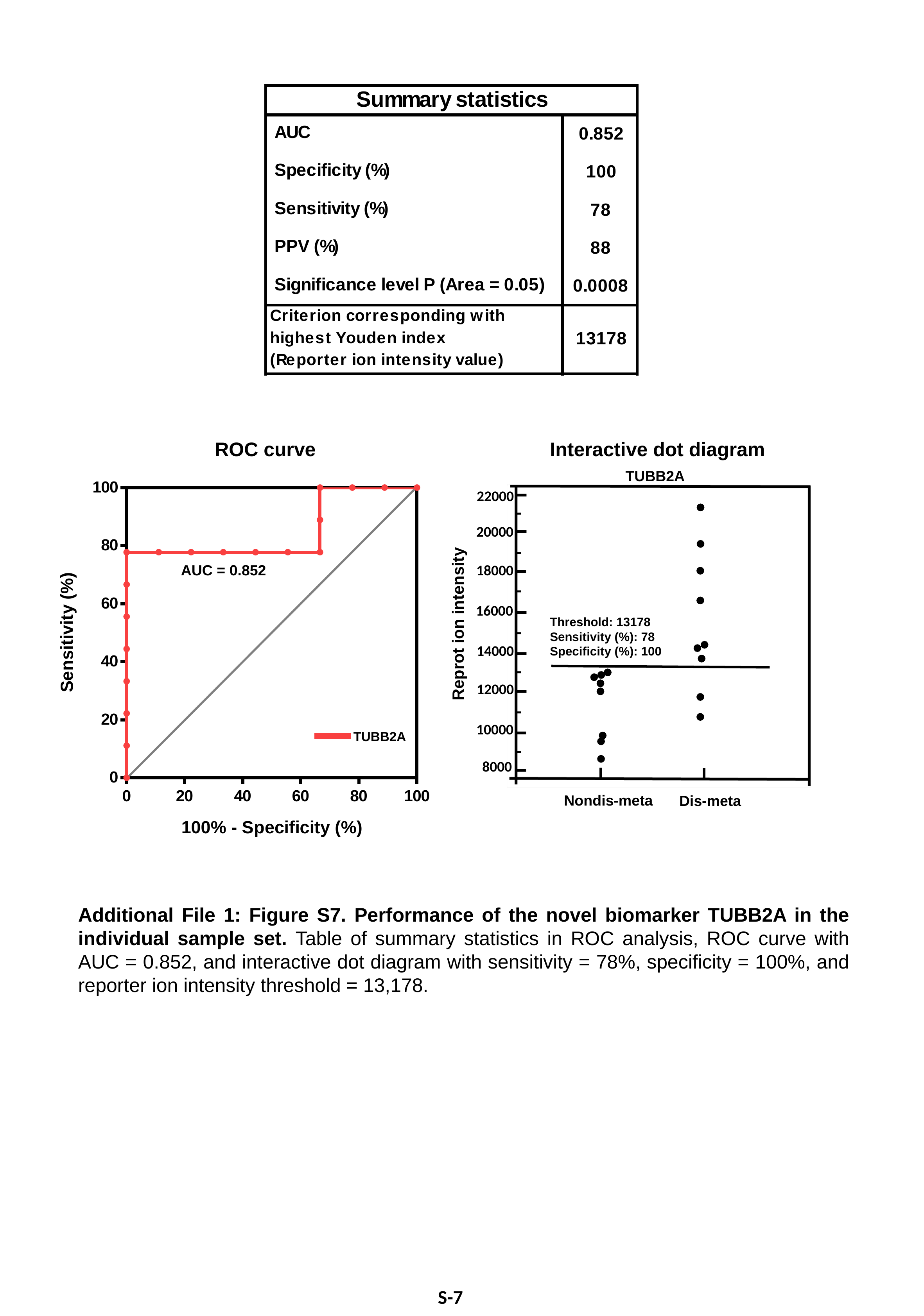

ROC curve
Interactive dot diagram
TUBB2A
22000
20000
18000
AUC = 0.852
16000
Reprot ion intensity
Threshold: 13178
Sensitivity (%): 78
Specificity (%): 100
14000
12000
10000
TUBB2A
8000
Nondis-meta
Dis-meta
Additional File 1: Figure S7. Performance of the novel biomarker TUBB2A in the individual sample set. Table of summary statistics in ROC analysis, ROC curve with AUC = 0.852, and interactive dot diagram with sensitivity = 78%, specificity = 100%, and reporter ion intensity threshold = 13,178.
S-7

## Slide 11
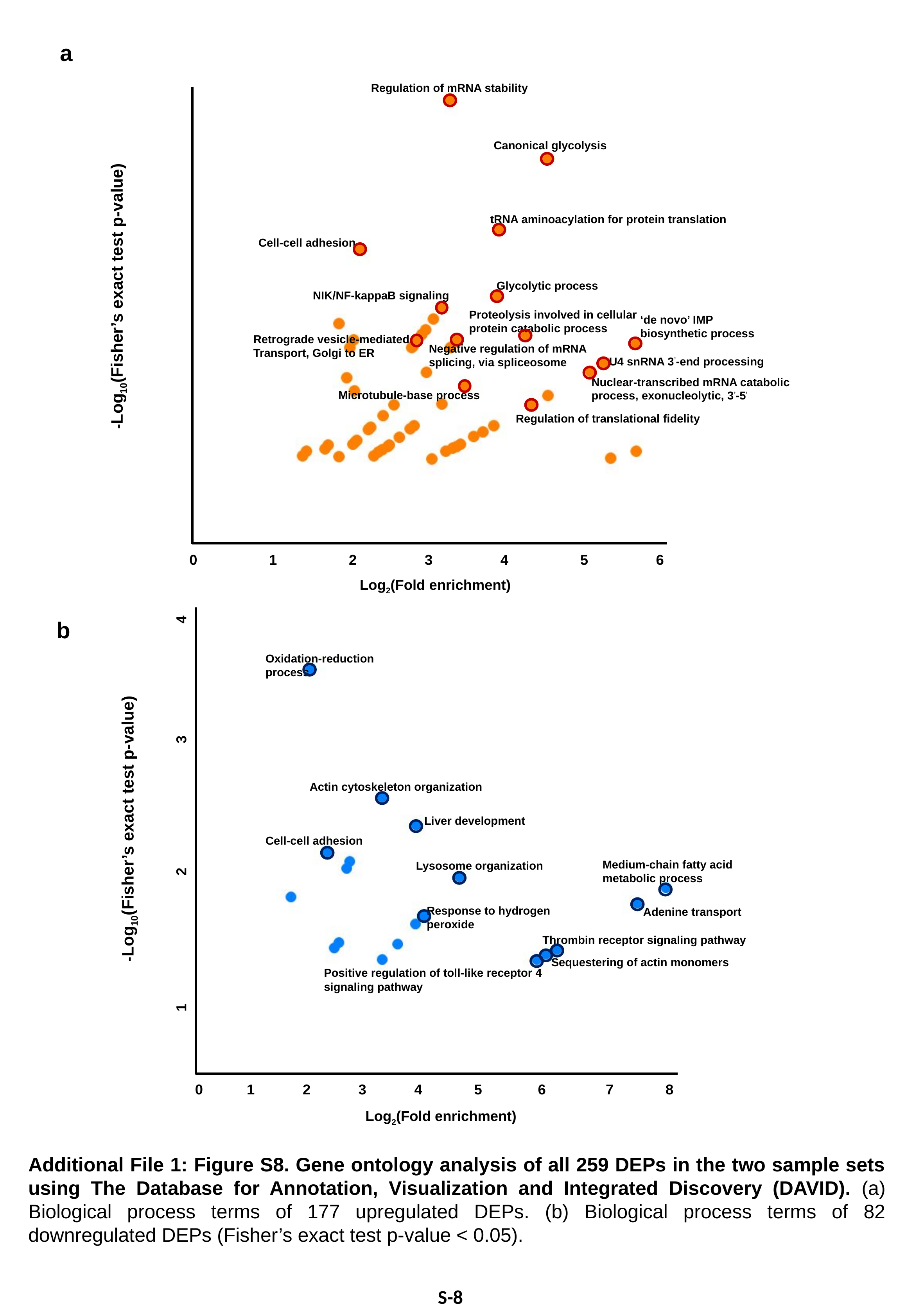

a
Regulation of mRNA stability
Canonical glycolysis
tRNA aminoacylation for protein translation
Cell-cell adhesion
Glycolytic process
-Log10(Fisher’s exact test p-value)
NIK/NF-kappaB signaling
Proteolysis involved in cellular protein catabolic process
‘de novo’ IMP
biosynthetic process
Retrograde vesicle-mediated
Transport, Golgi to ER
Negative regulation of mRNA splicing, via spliceosome
U4 snRNA 3’-end processing
Nuclear-transcribed mRNA catabolic process, exonucleolytic, 3’-5’
Microtubule-base process
Regulation of translational fidelity
 0 1 2 3 4 5 6
Log2(Fold enrichment)
b
Oxidation-reduction process
Actin cytoskeleton organization
Liver development
-Log10(Fisher’s exact test p-value)
Cell-cell adhesion
Medium-chain fatty acid metabolic process
Lysosome organization
 1 2 3 4
Response to hydrogen peroxide
Adenine transport
Thrombin receptor signaling pathway
Sequestering of actin monomers
Positive regulation of toll-like receptor 4 signaling pathway
 0 1 2 3 4 5 6 7 8
Log2(Fold enrichment)
Additional File 1: Figure S8. Gene ontology analysis of all 259 DEPs in the two sample sets using The Database for Annotation, Visualization and Integrated Discovery (DAVID). (a) Biological process terms of 177 upregulated DEPs. (b) Biological process terms of 82 downregulated DEPs (Fisher’s exact test p-value < 0.05).
S-8

## Slide 12
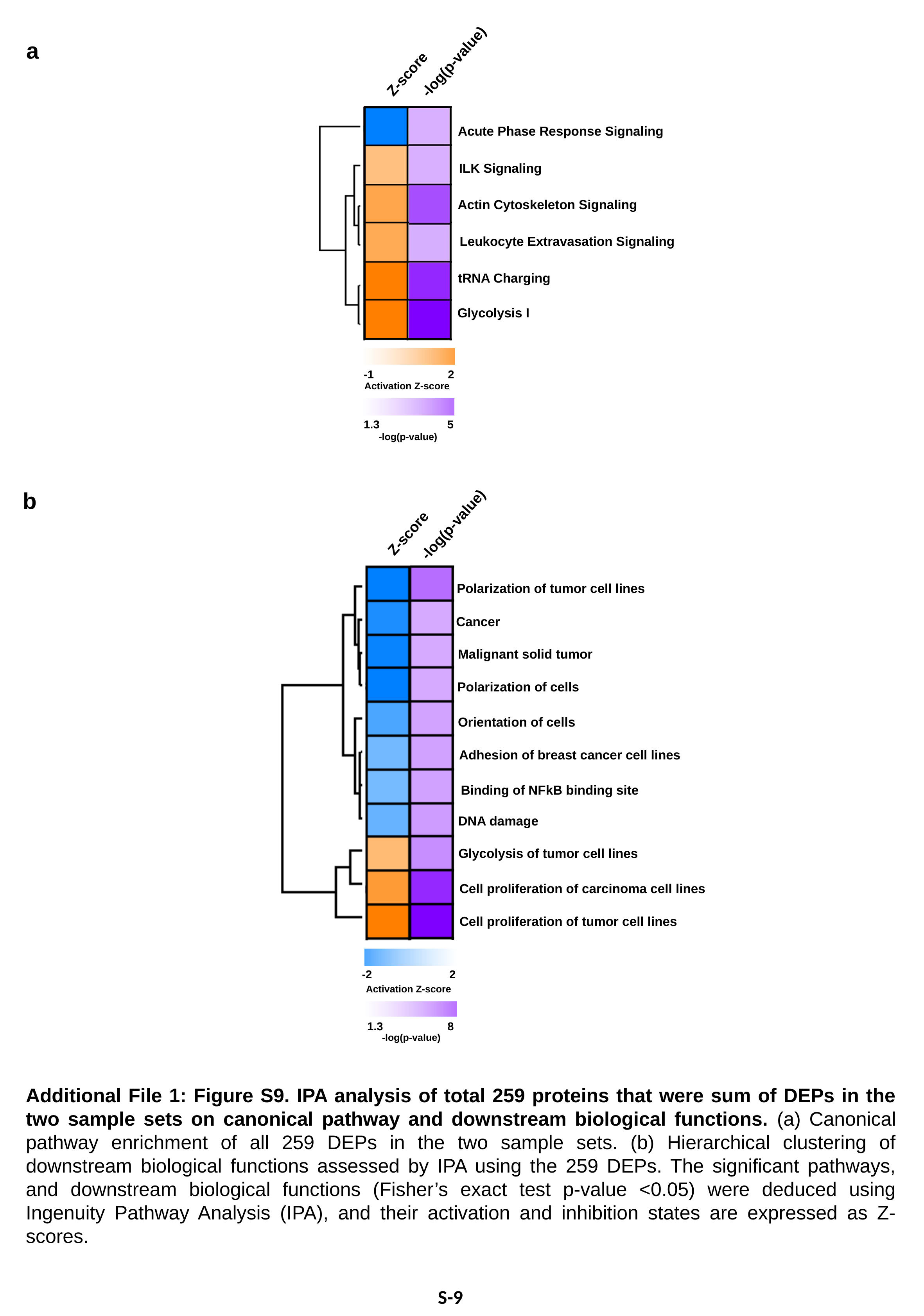

a
-log(p-value)
Z-score
Acute Phase Response Signaling
ILK Signaling
Actin Cytoskeleton Signaling
Leukocyte Extravasation Signaling
tRNA Charging
Glycolysis I
-1 2
Activation Z-score
1.3 5
-log(p-value)
b
-log(p-value)
Z-score
Polarization of tumor cell lines
Cancer
Malignant solid tumor
Polarization of cells
Orientation of cells
Adhesion of breast cancer cell lines
Binding of NFkB binding site
DNA damage
Glycolysis of tumor cell lines
Cell proliferation of carcinoma cell lines
Cell proliferation of tumor cell lines
-2 2
Activation Z-score
1.3 8
-log(p-value)
Additional File 1: Figure S9. IPA analysis of total 259 proteins that were sum of DEPs in the two sample sets on canonical pathway and downstream biological functions. (a) Canonical pathway enrichment of all 259 DEPs in the two sample sets. (b) Hierarchical clustering of downstream biological functions assessed by IPA using the 259 DEPs. The significant pathways, and downstream biological functions (Fisher’s exact test p-value <0.05) were deduced using Ingenuity Pathway Analysis (IPA), and their activation and inhibition states are expressed as Z-scores.
S-9

## Slide 13
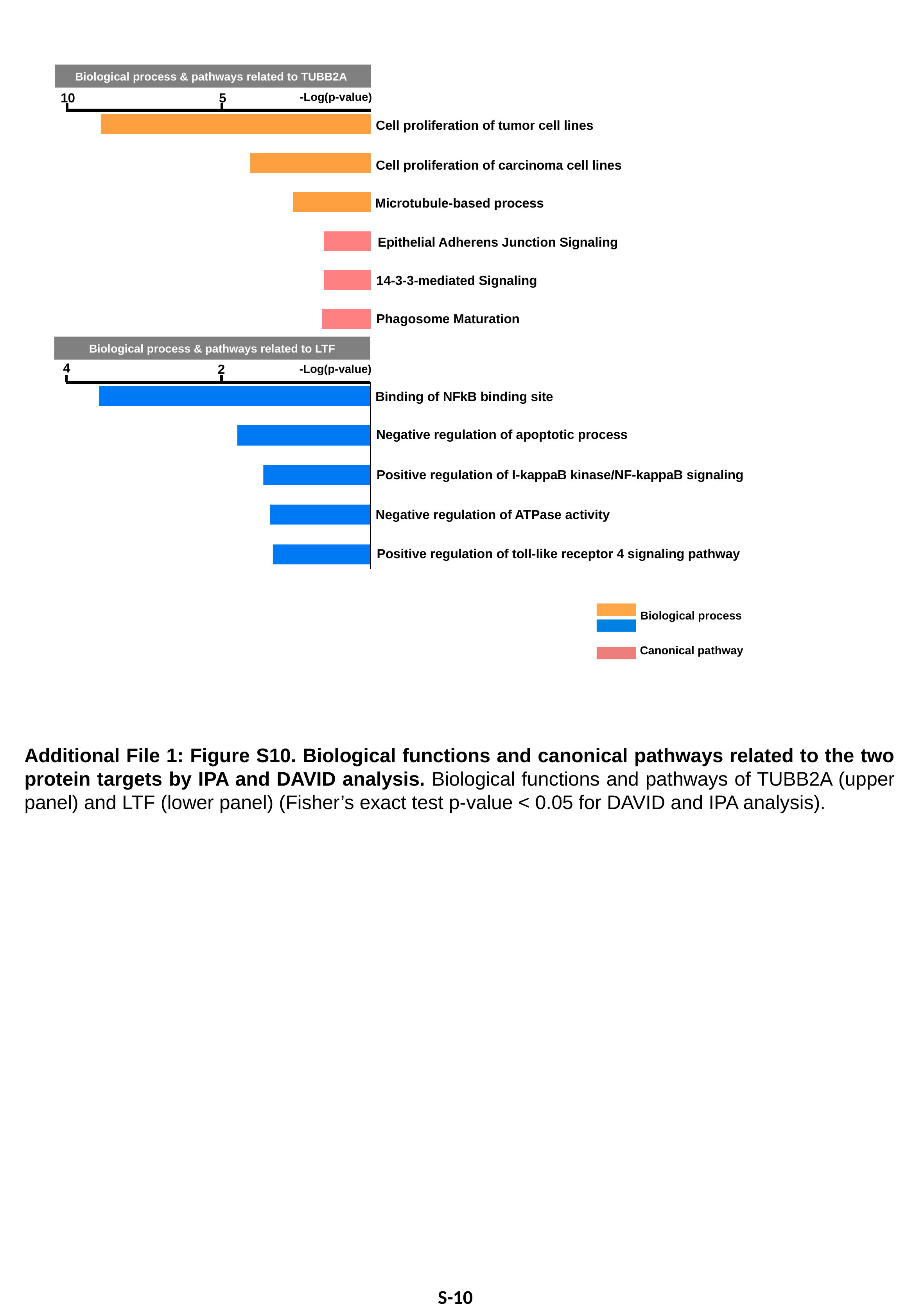

Biological process & pathways related to TUBB2A
10
5
-Log(p-value)
Cell proliferation of tumor cell lines
Cell proliferation of carcinoma cell lines
Microtubule-based process
Epithelial Adherens Junction Signaling
14-3-3-mediated Signaling
Phagosome Maturation
Biological process & pathways related to LTF
4
2
-Log(p-value)
Binding of NFkB binding site
Negative regulation of apoptotic process
Positive regulation of I-kappaB kinase/NF-kappaB signaling
Negative regulation of ATPase activity
Positive regulation of toll-like receptor 4 signaling pathway
Biological process
Canonical pathway
Additional File 1: Figure S10. Biological functions and canonical pathways related to the two protein targets by IPA and DAVID analysis. Biological functions and pathways of TUBB2A (upper panel) and LTF (lower panel) (Fisher’s exact test p-value < 0.05 for DAVID and IPA analysis).
S-10
